# Calling Sample Mix-Ups in Cancer Population Studies

**DOI:** 10.1371/journal.pone.0041815

**Published:** 2012-08-09

**Authors:** Andy G. Lynch, Suet-Feung Chin, Mark J. Dunning, Carlos Caldas, Simon Tavaré, Christina Curtis

**Affiliations:** 1 Department of Oncology, University of Cambridge, Cambridge, Cambridgeshire, United Kingdom; 2 Cambridge Research Institute, Cancer Research UK, Cambridge, Cambridgeshire, United Kingdom; 3 Department of Preventive Medicine, University of Southern California, Los Angeles, California, United States of America; Ohio State University Medical Center, United States of America

## Abstract

Sample tracking errors have been and always will be a part of the practical implementation of large experiments. It has recently been proposed that expression quantitative trait loci (eQTLs) and their associated effects could be used to identify sample mix-ups and this approach has been applied to a number of large population genomics studies to illustrate the prevalence of the problem. We had adopted a similar approach, termed ‘BADGER’, in the METABRIC project. METABRIC is a large breast cancer study that may have been the first in which eQTL-based detection of mismatches was used during the study, rather than after the event, to aid quality assurance. We report here on the particular issues associated with large cancer studies performed using historical samples, which complicate the interpretation of such approaches. In particular we identify the complications of using tumour samples, of considering cellularity and RNA quality, of distinct subgroups existing in the study population (including family structures), and of choosing eQTLs to use. We also present some results regarding the design of experiments given consideration of these matters. The eQTL-based approach to identifying sample tracking errors is seen to be of value to these studies, but requiring care in its implementation.

## Introduction

It is a truism that, whatever the care taken, if a study becomes large or complex enough then errors will occur in sample tracking. This issue has had a high profile of late following an error at a personalized genetic testing service (http://spittoon.23andme.com/2010/06/08/update-from-23andme/), problems uncovered by recent ‘forensic’ investigations of genomic scale studies [1], and the recent highlighting of errors in several high-profile studies [2]. In addition to these major problems, throughout the years of high-throughput studies, such errors have been nominated as the likely cause of discrepant results [3], [4]. Naturally, for some time, there have been calls to take care to limit such errors [5], and a number of strategies to reduce or detect errors are regularly used.

It is common to use replicated control samples at known points on a plate [6], which should pick up any major errors (although if these are in the same positions on each plate, then they will not highlight the wrong plate being used). In addition to this limitation, the expense of such an approach may make it unattractive. Many expression platforms offer the opportunity to mix external controls with the sample to be hybridized, and initiatives such as the External RNA Controls Consortium (ERCC) [7] can only be advantageous in this regard. Indeed the use of such controls has recently been demonstrated for Affymetrix GeneChips [8]. When genotyping arrays are being used (possibly for the purpose of inferring DNA copy-number) then we have a fundamental metric for identifying samples that will be of use if multiple samples are hybridized from the same individual [9], or if we have prior knowledge of genotypes [10].

Known phenotypes with a sole (or strong) genetic component can also be used to check sample validity (or rather to seek to detect plating errors - as they are unlikely to have enough power to confirm that a sample is that which it claims to be). Sex is the obvious phenotype in this regard. With a careful sample layout, as is discussed later, errors on a plate scale would be detected by a sex-check, but individual switches of any pair may not. Clearly for some studies, e.g. in prostate cancer, this will not be an option. Other traits such as blood group could be compared to the appropriate genotypes, but for a trait with a narrow driving locus there is too great a chance that there will be a miscalling of the genotype class simply to exclude samples based upon this metric. Many such traits are therefore needed.

Expression Quantitative Trait Loci (eQTLs) that regulate the transcript abundance of particular mRNAs can be identified systematically using high-throughput technologies [11] and can provide this large number of traits, with approximately 5% of genes showing cis-eQTL driven behaviour [12]. Many studies aim to infer eQTLs given a set of genotypes, a set of expression measurements and a mapping between the two. It follows then that given the genotypes, the expression measurements and a set of eQTLs one should be able to say something about the mapping. In short, given a set of expression arrays and eQTLs, one can make predictions as to the genotypes that one might expect to drive the expression and then seek to identify a genotype array that has measured similar values.

**Figure 1:**
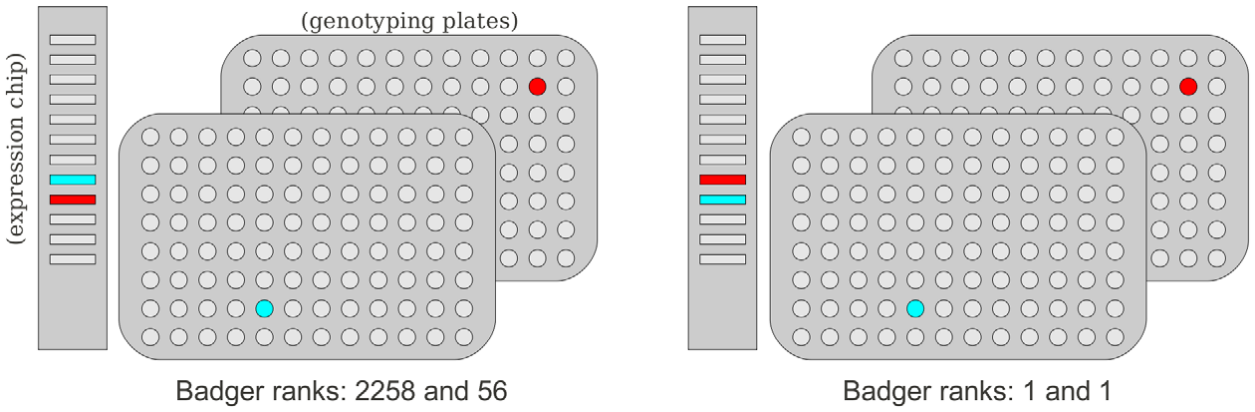
Example of a simple sample switch. One expression BeadChip (12 arrays), and two plates of samples for genotyping are illustrated. In particular, in the left panel, the intended locations of two samples are highlighted (in blue and red) for the two technologies. The BADGER ranks for the association between these two expression arrays and genotype arrays are high and indicate that there is a mis-mapping. On the right hand side the resolution to this example is shown. Not only with a simple switch can we match the expression arrays to the genotype arrays (now with BADGER ranks of 1), but since the two genotyping arrays are from different plates, while the two expression arrays are neighbouring, we can deduce that the error took place on the expression chip.

**Figure 2:**
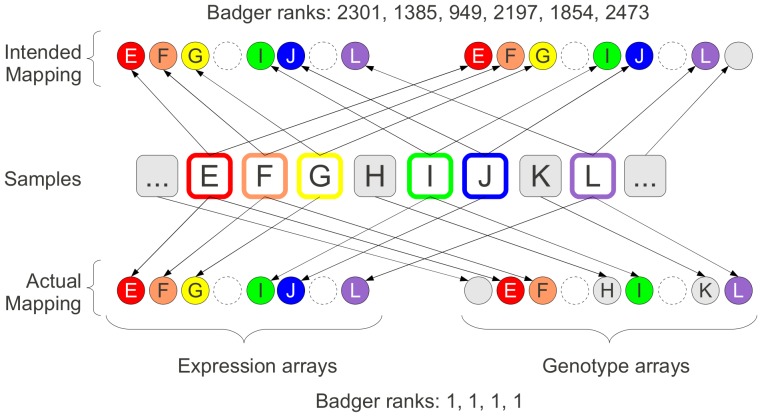
Example of a complex sample switch. A series of patients (referred to by letter) from whom samples are taken (middle row, samples depicted as squares) are seen at some point. Some time later, six of these patients (E,F,G,I,J,L) satisfy the criteria for inclusion in a retrospective study and it is intended to run the samples from those patients on expression arrays (circles, top-left) and genotype arrays (circles, top-right). Since the patients (and thus samples) formed a sequence, we include space-holders in the depiction of the arrays for those samples that were not suitable for the study in question (denoted by dashed circles for the arrays and grey shading for the samples). The BADGER ranks for the expression and genotype arrays that were supposed to be associated with these six samples range from 949 to 2473 suggesting that not one of the six is correctly mapped. The resolution is difficult to find unless one knows about the original sample sequence, including the samples that are not part of the retrospective study. When resolved (bottom row) we see that the samples going onto the genotype arrays have ‘slipped’ by one position with the result that samples G and J have been run on expression, but not genotyping arrays, while samples H and K (which were not meant to have been run at all) have been run on genotype, but not expression arrays. For the four arrays that have been run on both technologies, we can see that the BADGER ranks are now perfect. It is worth noting that the expression arrays on which samples G and J were run have a high ‘minimum BADGER score’ which is a sign that the sample does not feature on any genotyping array in the study.

The ability to predict SNPs from expression data has recently been considered from a data-security context [13], but it is an approach we have used to prospectively ensure data integrity in the Molecular Taxonomy of Breast Cancer International Consortium (METABRIC) study [14], and have applied to other cancer studies. The approach we termed BADGER (“Bead Array Diagnostic for Genotype and Expression Relationships”) and it is described in the Methods section. Examples of two situations (one simple, one more complex) where confusion over sample identity has arisen in large-scale studies, and been resolved by BADGER, are given in Figures 1 and 2.

Our approach is conceptually similar to the recently published MixupMapper approach to this problem [2], but differs in implementation due to the nature of the data to which we apply it. It also differs in the choice of implementation environment, with BADGER being developed in R [15] and MixupMapper in Java. In particular, Westra and colleagues’ approach looks to find the best expression match for a genotype array, while ours has been to look, in the first instance, for the best genotype match for an expression array. It is generally easier to identify duplicate genotype arrays than duplicate expression arrays and thus we can ensure, if we wish, that we are mapping towards a set of unique arrays.

Both approaches rely on defining a distance between an expression array and a genotyping array. Our measure (the ‘BADGER score’) is the sum (across eQTLs) of the squared difference between the number of ‘B’ alleles called from the genotype array and the number predicted from the expression array. MixupMapper, on the other hand, uses a normalized sum of z-scores for the difference between predicted and observed expression values. As a minor additional detail, MixupMapper considers the magnitude of their score, while BADGER looks at the rank. Naturally, a low score is indicative of a match. The vast majority of arrays (ideally all but one) will not be a match, so the score of an array that does match should be outlying and take a rank of one.

As the concept of identifying plating errors using eQTLs has been demonstrated, we will not focus on justifying or demonstrating the approach once more (although a few such results are presented). Rather, we shall highlight the challenges of applying such an approach to a population cancer genomics study, and note where the distinctions of our take on the approach lend themselves to such data.

## Results

While our primary purpose is not to demonstrate once more that an eQTL-based approach to calling and identifying mismatched samples can work, we note that our results here would support the message of Westra and colleagues [2]. We instead seek to highlight some of the factors that can lead to misinterpretation of the results of an eQTL-based approach to identifying mismatches when applied in large tumour studies. Specifically, we will consider the effects of the loss-of-heterozygosity (LOH) and departure from diploid status that we expect to see in tumour samples, the impact of cellularity, and the consequences of having a mixture of ethnicities in a study. In addition, we will report how study design affects our ability to use such an approach.

### Calling Mis-mappings with Tumour Samples

Westra and colleagues [2] note that it is possible to “identify genotypes that clearly did not match any gene expression arrays”. With prospectively obtained collections of normal tissue this would seem to be the case, but with retrospective studies of tumour tissue, a number of additional problems have come to light. Most obvious is the fact that these methods expect to see diploid genotype calls, and the tumour samples may be anything but diploid (although many algorithms will still generate diploid genotype calls from these samples). Then there is the issue of stromal contamination of the tumour samples. This may, in the sample from which DNA was extracted, be at a different level from that in the sample from which RNA was extracted. Finally, there may be a mutation within the tumour that disrupts the biology driving the eQTLs on which our tests are based.

**Figure 3:**
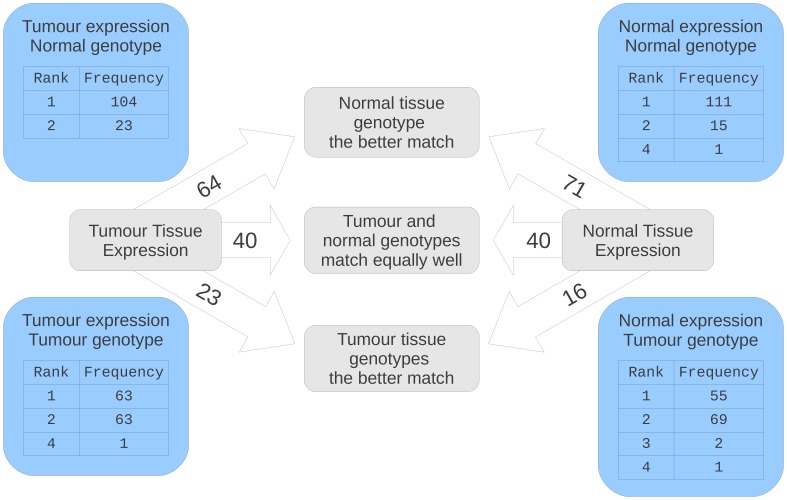
The effect of tumour expression and genotypes on apparent matching quality. For 127 quartets of matched tumour-and-normal genotype-and-expression arrays we illustrate the relative quality of the matches between the four different expression-genotype combinations. The 127 genotype array pairs are all clearly well-matched (not shown). Our approach is to identify the quality of a genotype array’s match to an expression array, and all results given are reflective of this direction of comparison. For the sets of tumour and normal expression arrays, indicated in the arrows are the number of expression arrays for which the two genotyping arrays are equally good matches (have the same genotype calls), the number for which the tumour genotyping array for that sample has a better score than the normal genotyping array, and vice versa. Additionally, in the corner panels, the BADGER ranks for the matches amongst the entire data set to which we have access are presented.

For the 127 samples for which all four arrays (SNP/expression for tumour and normal tissue) are available, the qualities of the matches from normal and tumour tissues are shown in Figure 3. In general, for both normal and tumour expression arrays, the ‘normal’ genotype array proved to be a better match than the ‘tumour’ genotype array.

The concern then is that, in circumstances where we have not processed the normal genotype, the tumour genotype would sometimes not be a good enough match to allow us to assign the match correctly. Anecdotally, this does seem to occur. A possible explanation is that the non-diploid (or diploid but suffering from LOH) nature of the tumour genotype interferes with the calling of diploid SNPs. If the majority of the SNP/expression relationships observed are not directly causally linked, then this would explain the relatively poor match qualities of tumour SNP arrays as compared to normal tissue SNPs.

### Cellularity and RNA Quality

Since the normal genotype array is shown to be a better match than the tumour genotype array, even though our eQTLs were defined mainly from tumour samples, it seems reasonable to suppose that cellularity (the contamination of tumour tissue with stromal tissue) will have little effect on the performance of approaches such as BADGER. Normal contamination will increase the chances of calling heterozygous SNPs, even when the tumour has undergone LOH or allele-specific DNA copy-number changes.

Although the disrupted genotyping calls due to the copy-number aberrations in tumours can impede approaches such as BADGER, these remain an accurate description of the tumour and we must endeavour to identify the sample and match it to an expression array. As has previously been noted, a poor quality expression array can also disrupt the process [2], but the quality of an array is estimable and can be compensated for, or the array can simply be discarded (at least when defining the eQTL relationships).

**Figure 4:**
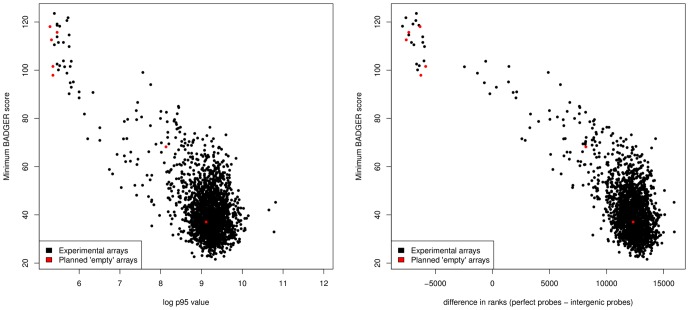
Expression array quality scores and association with BADGER performance. In order to illustrate a wide range of array qualities, this figure includes some poor quality arrays that (for this very reason) were excluded from METABRIC. Left panel: Illustrating the association between the 95th percentile of observed log-intensities (p95) and the minimum BADGER score associated with the array. Right panel: the association between 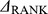 and minimum BADGER score. Also indicated, in both cases, are the arrays where no sample was scheduled to be hybridized.

Figure 4 shows the association between two measures of array quality (see Methods) and the minimum BADGER score associated with an array (an indicator of whether a matching SNP array could be found). Both statistics are good predictors of the performance of an array, with the 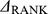 statistic doing better than P95. Clearly at least one of the arrays scheduled to be empty actually had a sample hybridized to it, and a number that had samples assigned to them failed to hybridize. Note that when there is no signal on an array, the rank difference is not zero as one might expect, but rather it is substantially negative.

### Ethnicities

Using principal component loadings published for the shellfish tool (www.stats.ox.ac.uk/~davison/software/shellfish/shellfish.php), one can project Affymetrix SNP data onto a triangle where the three corners represent the HapMap populations from which the loadings were derived. For convenience we will term the groups of samples that form in these corners the ‘Europe group’, the ‘Africa group’, and the ‘Asia group’. We have also observed individuals lying between the Africa and Europe groups (whom we shall combine in the ‘Africa/Europe group’) and between the Europe and Asia groups (whom we shall combine in the ‘Europe/Asia group’).

**Figure 5:**
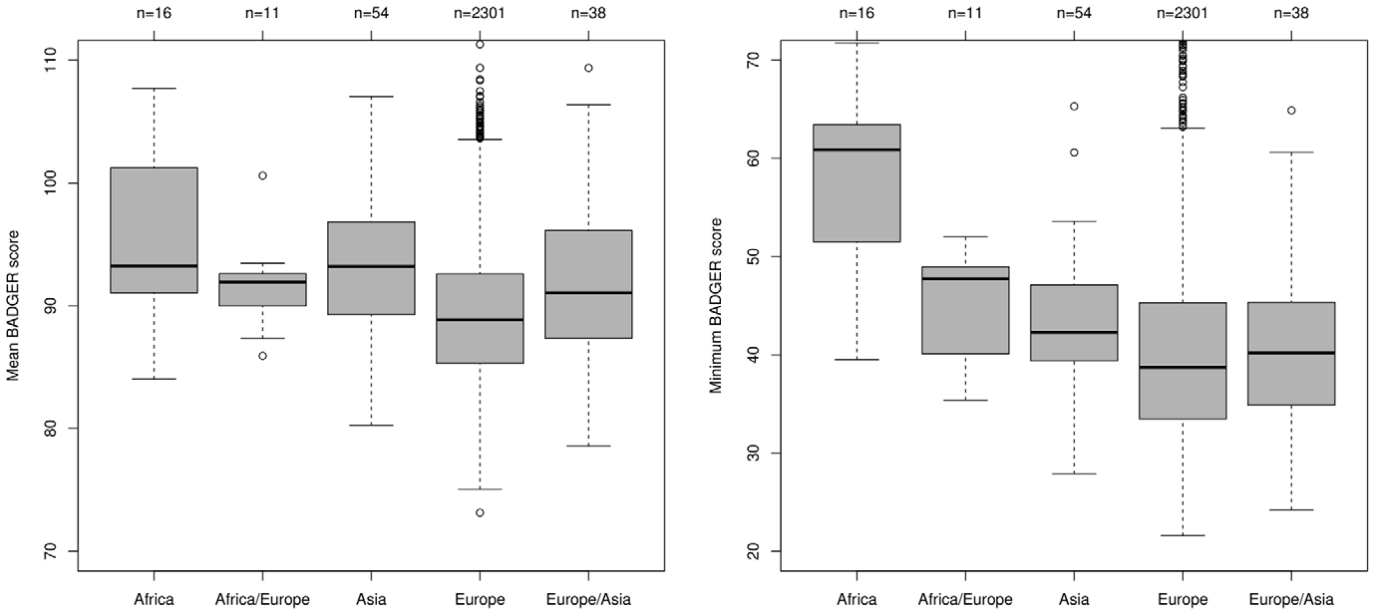
The effect of ethnicity on BADGER performance. Every genotyping array is compared to every expression array, and a score assigned to the match (the lower the score the better the match). In the left hand panel, the mean score by genotype array is compared to the ethnicity of the patient as inferred from the genotyping array. In the right hand panel the minimum score associated with a genotyping array (a better indicator of whether a match exists) is plotted by ethnicity.

We plot the average BADGER score (NB score not rank) associated with each genotype array against group in the left hand panel of Figure 5. We see that the mean score is lower in the Europe group. This is to be expected, as the Europe group contributes the vast majority of expression arrays in our collection and one would not be surprised that these may predict genotypes that are more similar to those obtained from other samples in the Europe group. Also, being in the majority, the Europe group patients drive the eQTL-like associations used by BADGER and these associations may differ between the groups.

More important than the average score is the minimum score that, for any genotyping array with a matched expression array in the data set, we might expect to be comparable regardless of ethnicity. In the right hand panel of Figure 5 we see that this is not the case, and that while most groups are indeed comparable, the Africa group exhibits minimum scores that are higher. We have no *a priori* reason to believe that this group will be over-represented in the number of genotyping arrays for which no matched expression array exists. For a relatively high proportion of this group we cannot be sure of the match between genotype and expression, but this is more likely to be a consequence of the higher scores seen in the Africa group than a cause of it.

**Figure 6:**
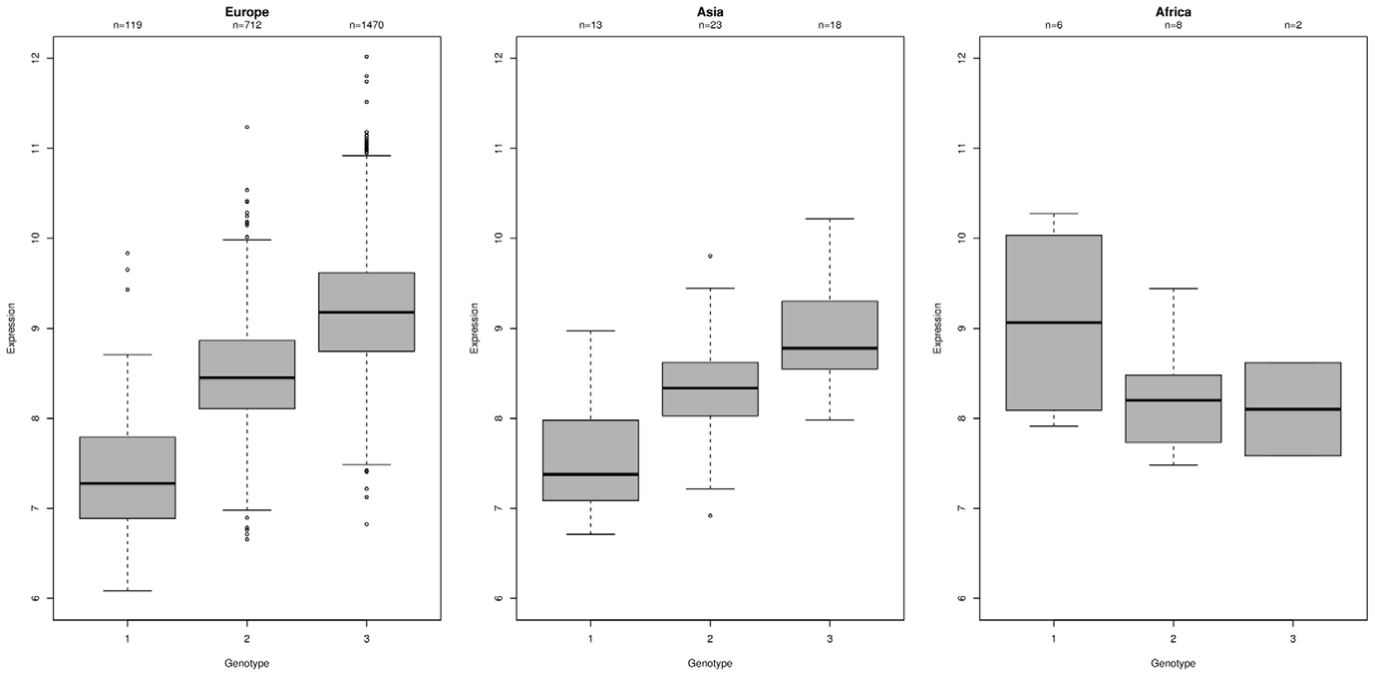
Example of eQTL behaviour differing by ethnicity. Depicted are the log-intensity values for the Illumina ILMN_1710752 probe in the NAPRT1 gene plotted against the genotype calls for the rs10112966 SNP from the Affymetrix SNP_A-4292499 probe (all in the 8q24.3 region of the human genome). Naturally only data from those genotyping and expression arrays that can be matched are shown. The association is shown for three groups. The association between this SNP and gene has previously been noted [26], as have the differing allele frequencies between groups.

We cannot claim, for any eQTL pair we use, that the genotype we observe is actually driving the expression (see next section). At best it is likely to be a tagging SNP for the causal variant (if one exists), and the performance of the tagging SNP will vary between ethnic groups. It has been observed that only 50% of eQTLs are seen in more than one population, and a very small minority in several [16], [17]. Thus it should not surprise us to see behaviour such as that shown in Figure 6 where the association between genotype and expression that is so clear in the Europe and Asia groups is not evident in the Africa group. Since this group is in the minority, the predicted genotypes generated for these individuals from an association defined by the Europe and Asia groups will be poor at best.

### Close Relatives and Validation

To illustrate further aspects we consider an additional data set: the genotype and expression data associated with the HapMap (Phase I) samples [18], as originally studied by Stranger *et al*. [16], and used as one of the illustrative datasets in the MixupMapper paper [2]. While not a cancer study, this allows us first to confirm that BADGER can identify the problems that MixupMapper identified, second it allows us to examine a dataset with known family structures, and third it provides an illustrative study with greater balance of ethnicity, balance of sex, and presumed better quality of data since this was a prospective study. Finally, it provides a more useful data set for investing the ‘power’ of these techniques. See Sweave S1 for full details.

MixupMapper identified only one mix-up in this data set, finding that the best expression match for the genotype data supposedly from sample NA18515 was that supposedly from sample NA18517.

In the CEU population, there is a problem with one of the expression arrays associated with "NA10856" (labelled GSM232786_NA10856_2_2). However it is clear that the other three expression arrays are good matches, and with some investigation, it is apparent that the values for this array are identical to one of the Yoruban arrays (labelled GSM232802_NA18503_1_1), but that the values differ in GEO, making it unclear where the problem arose. We can speculate that since MixupMapper would have found a good match for the NA10856 genotype array, that it would not have flagged this as being problematic.

With BADGER, we also find that the four expression samples associated with NA18515 all offer the genotype array mapped to NA18853 as the best match. It is true that the expression arrays associated with NA18517 are the best matches for the SNP array mapped to NA18515, however this is because NA18517 is a parent of NA18515. The expression arrays associated with NA18516 (the other parent) are the next best match. In the absence of an expression array for the child, we would expect the best match to be one of the parents. In fact, we can easily see from the genotypes of the mother/father/child trio that the error (if there is an error) must be in the expression array.

The genotype array that offers the best match to all of the NA18515-associated expression arrays is that associated with NA18853. The match is marginally worse than that for the expression array associated with NA18853, but noticeably better than that associated with NA18854 (the child of NA18853). Comparison of the full expression profile suggests that the NA18515 expression arrays are not simply accidental replicates of NA18853. Thus the mix-up is difficult to resolve from such a distance, and we would recommend removing the expression arrays associated with NA18515 from analyses but would leave the genotype arrays as they are. Note that we are using the expression matrix given by Westra *et al*. so can make no claims regarding the original study [16].

While the match to a close relative tends not to be as good as that to the correct sample, we do see enough overlap in values that if one of the samples were missing then we would be in danger of mistakenly associating the two as being from the same individual. This would presumably be more of a danger if the genotype array were missing as the existence of relatives will be harder to deduce from the expression data.

Taking the Han Chinese and Japanese individuals, as these do not contain complicating family groups, we can simulate sample switches and confirm the utility of BADGER. We find that half of the samples have to be switched to require more than one productive iteration (the final iteration is always to confirm that there are no more switches to make), and two-thirds must be switched for there to begin to be unresolved switches. This may be an over-estimation of performance, since our ‘external’ eQTL set was, in fact, defined by Westra et al. from these data, but competing against this are the lack of subtlety in the corrections that in practice is afforded by the human assessment of the potential switches and lab-validation between iterations. The assessment here was simplistic and automated for the simulation study. Even allowing for these points, the fact that 80 of the 90 samples would need to be deranged before an approach such as BADGER is unable to add value is remarkable.

### Choice of eQTLs

Defining the eQTLs from the data set therefore can potentially impact upon any genetically distinct minority group in a study. Westra and colleagues [2] mention some of the potential benefits of using externally defined eQTLs. There would certainly be benefits to doing so if we could ensure that all subgroups were represented. It is known that the performance of expression probes can be affected by SNPs [19] that happen to be covered by a particular probe and that this phenomenon can mimic eQTL behaviour (‘cis-eQTL artefacts’ [20], [21]). We have shown specifically that this is a problem for the longer probes of Illumina BeadArrays [22].

Many eQTL studies separate out such expression probes in order to avoid spurious associations [23]. We would suggest that these ‘cis-eQTL artefacts’ not only assist in the process as noted by Westra *et al*., but may provide a robust basis for correcting errors. By exploiting a technical artefact in this way, we would hope to be less sensitive to genuine biological differences between groups of patients than we would be if relying in uniformity of genuine eQTL behaviour across populations.

We initially chose a set of 383 eQTLs on the basis of the strength of association seen in our data. The significance of the association is as much a measure of the distribution of genotypes as it is the discriminatory power of the expression-SNP association, but this is a first pass and the set is refined as part of the BADGER approach as detailed in the Methods. Naturally some of these eQTLs are ‘cis-eQTL artefacts’. Indeed, this initial set of expression probes is already enriched for probes that cover SNPs with 184 out of 383 (48%) falling into this category as opposed to 11,027 out of 34,361 (32% ) of reliable probes on the array. Full details of SNP coverage for Illumina expression arrays are given in Table 1. Similar information is available for Affymetrix arrays [24].

**Table 1:** Numbers of SNPs in reliable probes for Illumina Human expression BeadArrays.

Generation ofBeadArray	0SNPs	1SNP	2SNPs	3SNPs	4 or moreSNPs
fourth generation	23454	8016	2060	517	429
third generation	23334	8188	2031	461	347
second generation	21734	7262	1680	364	239
first generation	16207	6786	1966	495	326

From the annotation packages in Bioconductor 2.9, for four generations of Illumina expression BeadArray (illuminaHumanv3.db, illuminaHumanv3.db, illuminaHumanv3.db, and illuminaHumanv4.db), noting the number of expression probes annotated as being good or perfect and covering 0, 1, 2, 3 or ‘4 or more’ SNPs.

When the set of expression-SNP associations is reduced to 125 probe-pairs, it is further enriched for SNP-covering probes. Only 26% of the 199 probes that do not cover SNPs survive into the refined set, while this increases to 33% for the 119 probes that cover one SNP, 47% for the 38 probes that cover two SNPs and 59% for the 27 probes that cover more than two SNPs.

**Figure 7:**
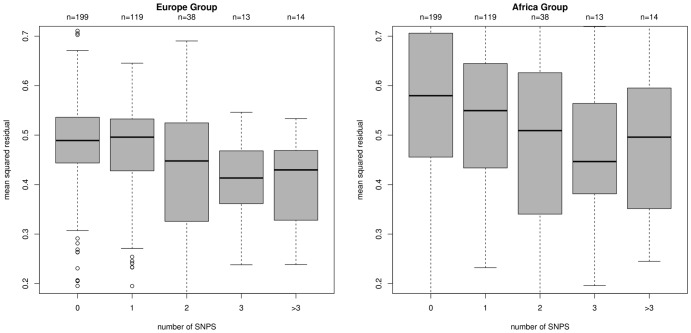
The coincidence of expression probes and SNPs, and the magnitude of residuals. Plotting for the set of 383 eQTLs, the mean squared residuals (predicted - observed B-allele counts) against the number of SNPs that are situated ‘under’ the expression probe according to the annotation. On average, the predictions are closer to the observations for probes that lie over multiple SNPs.

To reinforce this point, amongst our set, there is a trend that the more SNPs the expression probe covers, the smaller the discrepancies between the predicted and observed values of the SNP-probe in the eQTL association. This is shown for the Europe and Africa groups in Figure 7. Our eQTLs are biased towards the Europe group that contains the majority of our samples and so the observed associations are stronger for this group. Despite associations in the Africa group being generally weaker, the probes that cover multiple SNPs still explain a useful proportion of the variation of log-expression and if chosen in an unbiased way (e.g. solely based on annotation) they may provide a set of probes that will be reliable across the different population groups.

## Design of Experiments

### Plate Layout for Sex-based Diagnostics

By choosing distinct but differing patterns, by sex, for the sample layout on a plate, and ensuring that the patterns do not have rotational symmetry, the plotting of the inferred sexes of samples by plate will provide a clear and definitive diagnostic as to whether the correct plate has been used and whether in the correct orientation. Individual, simple, switches of neighbouring samples (in column or row) may not be picked up, but we can maximize the chances of doing so by choosing patterns under our constraints that minimize the numbers of neighbouring pairs (in columns or rows) of the same sex.

**Figure 8:**
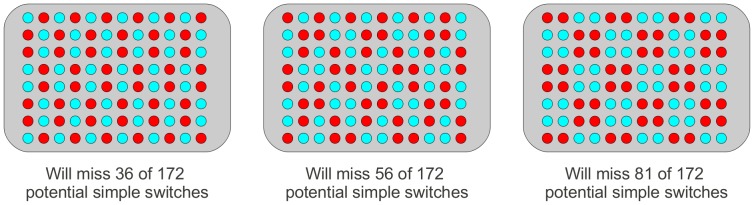
The ability of different layout designs to detect simple switches of neighbouring samples. Illustrated are three different patterns of sex (indicated by colour) by which samples could be laid out. There are 172 different sets of neighbouring pairs (ignoring diagonals) that one can identify in a 8×12 plate, and so 172 opportunities for a simple switch of neighbouring samples. For the simplest approach illustrated, 36 of these (three in each column) are of the same sex and so we would not be able to use sex as an identifier to spot the switching of these samples. The inverse of this design will be identical under rotation to the illustrated design and so it may not be desirable to use both. Thus we quickly see the need for more complicated designs such as the second illustrated here. Several permutations of the four basic columns will produce acceptable (and distinguishable) designs, with some small effect on the number of sample switches identifiable by the design. The third design shows how a possibly appealing layout, from the point of view of convenience, has poor ability to detect simple switches of samples - barely doing better than a random layout. Note that the 1–2–2–2–1 pattern of rows in the first and third examples is essential to avoid the rotational symmetry that would be present if we used a 2–2–2–2 pattern.

If the numbers of the sexes are equal in a study, a chequer-board pattern minimizes the numbers of neighbouring pairs of the same sex, but has rotational symmetry. Small perturbations from the chequer-board pattern will be susceptible to confusion (either to themselves via a rotation and small number of switches), or to one another (via slightly more switches). It seems likely that a regular pattern will be preferable, for ease of setting up the plate, and if only a small number of plates are required then this may be feasible. Note though that in order to avoid rotational symmetries, and indistinguishable plates, the number of simple switches on a plate that cease to be detectable (i.e., the number of pairs of neighbouring samples of the same sex) increases rapidly (shown in Figure 8).

If we had 100 plates on which the layout of the sexes was random, then by simulations we expect the closest pair of plates to differ in 30 wells (95% CI: 26 to 32), which would require a minimum of 15 simple switches to explain. Thus a random arrangement of the sexes will allow for the detection of plating errors. We would expect, with a random layout, to miss approximately 85 (95% CI: 72 to 98) of the potential simple switches on a plate (as opposed to at least 56 for all but the most trivial patterns of regular layout). Thus if the complication of attempting to use one of these regular designs was to come at the cost of doubling the probability of making a simple switch, then we might not expect it to bring benefits over a random layout. In such circumstances, if the natural ordering of samples would provide an ‘effectively random’ ordering of the sexes, then employing it is a highly defensible approach.

### Plate Layout for eQTL-based Diagnostics

If using eQTLs to identify our mismatches, in a scenario where only one sample per person is run on any technology, then whatever our design, we will always be able to detect a simple switch between samples run on neighbouring arrays. Once this is observed we can correct the recorded association between the genotype and expression arrays. If, however, we have additional sample information (and we usually will), the question may still remain as to which clinical sample is associated with each pair of arrays (this may not matter for some studies). If the two samples were neighbouring on the expression platform, but in completely different batches for the genotyping platform, then the confusion can be attributed to the expression platform and rectified (and *vice versa*). If the samples were neighbouring on both platforms then it may not be possible to identify the source of the error and so not subsequently possible to correct it.

Thus, in contrast to the case where sex was used to identify mix-ups, the natural approach (in this case to use the same layout for genotyping and plates) is not acceptable. Rather there is a desire then to have designs for the layout of the two experiments (expression and genotyping) that avoid (in general) neighbouring samples in one experiment being neighbouring samples in the other. This divergence of layout would ideally happen at the earliest possible stage in sample preparation. However, as with sex-based detection, there is a concern that adopting such an approach would enable us to detect plating errors at the expense of increasing their number. One might argue that, if all such errors were simple switches, then the success rate seen for resolving them in our approach and that of Westra *et al*. would prevent this from being a cause for concern. There are other errors such as contaminating two samples, omitting a sample or introducing an alien sample, from which we could not so easily recover, and which would caution us against such an attitude.

There is no approach that will be optimal for all combinations of study and laboratory. Each study group should then seek to balance these tensions as best they can within their own circumstances. What is clear though is that, for the successful resolution of mix-ups, complete layouts of samples at all stages of an experiment are required whether those layouts are ‘designed’ or not.

## Discussion

What is apparent from these results is that when dealing with retrospective clinical studies, there may be legitimate reasons why a genotyping array will not appear to be a match for an expression array even if material from the same person were run on both. Therefore, if one discards arrays solely on the basis that they do not appear to match, then there is a risk that one is discarding well-matched arrays. Moreover, these might be the really interesting samples where something unusual and revealing is happening. The importance of a predictor for the performance of BADGER is also clear. We do not wish to expend energy pursuing an apparent mismatch when the results have been driven by array quality.

Ideally then, we should discard arrays not because they do not appear to match, but because we can identify (possibly even validate) the error that led to the mismatch and yet not a way to correct for it. If we cannot identify the error then there are risks both in retaining the arrays (we may contaminate the study with erroneous data) or in removing them (by artificially making the study population more homogeneous we may compromise the external validity of our inferences).

If there is a distinct genotypic subgroup forming a small minority of samples in the study, then we have seen that these are particularly prone to being called as mismatches in error. Depending on the nature and purpose of the study, it may be that it makes sense not to include this subgroup in a primary analysis. Studies are often designed with inclusion criteria that restrict the heterogeneity of samples (sacrificing, in part, the external validity of the study for increased internal validity), and this could be seen in that context. If this is the view, it may make little practical difference if the arrays are excluded on the basis of the apparent mis-mapping of samples, or if they are excluded because they represent a minority pattern, in which case the policy of removing unresolved samples might be more attractive.

Had our purpose been to search for eQTLs, then removing the Africa group from analyses would undoubtedly have increased the number of ‘eQTLs’ that we would have found, but this can only be reasonable if we can recognize the restrictions on the interpretation of our findings that would result. In this example, it is clear what is happening, and it seems likely that we could relate our findings to a valid external population. Matters may not always be so clear, and the removal of samples on the basis of genetic patterns (when an actual error cannot be identified) will always have the potential to bias downstream analyses. A pragmatic approach may be to perform multiple analyses with different inclusion criteria in order to check the robustness of the results.

Crucial to this process is the existence of a set of eQTLs. These do not need to be of ‘publishable’ standard, and indeed their significance is not the same as their predictive power. One has the choice of using an independent data set to define these (as did Schadt et al. [13]), possibly exploiting an online catalogue of eQTLs. An alternative is to use a subset of data for which one has particular confidence, such as we have done. Finally, the definition of pseudo-eQTLs by considering the technologies being used may provide the required set. If none of these is available, then defining eQTLs from the full data set is still likely to work and, so long as the results are treated with appropriate caution, will do no harm.

Throughout we have referred to a process of working from the expression array to identify a matching genotype array. We propose working in this direction for a number of reasons. Firstly, it is easier to identify duplicate arrays (or arrays otherwise from the same sample) in the genotyping arrays and limiting the search to a set of unique arrays can be beneficial for interpretation of the BADGER results. If a genotyping array’s supposedly matching expression array was not the array with best BADGER score, it might not be as easy to identify that this is because an accidental duplication of that array has occurred. From an expression array’s point of view, since genotype duplicates can easily be removed, the supposedly matching genotype array should always have a BADGER rank of 1.

Additional beneficial factors include the greater consistency of quality between the genotype arrays, an easier to define metric for distance between genotypes than between expression values (some eQTLs, while providing good discrimination, see much greater variance of expression with some genotypes compared to others), and a possibly lower chance (in our experience) of plating errors occurring in the genotyping arrays than in the expression arrays. If, however, we were using externally defined eQTLs, then we may prefer to work from the genotype arrays towards the expression arrays as the quantification of the eQTL association may be dependent on factors, such as ethnicity, that we can infer only from the genotyping arrays.

In determining what error may have occurred it is important that we can access all information about a sample’s location at various stages in its preparation. Because not all samples are eligible for inclusion in a study, the ordering/layout of extracted RNA/DNA may not match the ordering/layout of the samples. In turn, perhaps to accommodate control samples or repeats of earlier failed arrays, the processed RNA/DNA may have a different ordering/layout again. Finally, if using a BeadChip with multiple arrays, the layout may change once more (hence the increased risk for the expression arrays mentioned above). If for the RNA and DNA we have access only to the final layout information then we may miss the source of an error or attribute it to the wrong experiment (e.g. RNA rather than DNA), which would be worse.

It would be preferable to minimize the number of plating errors, or to increase the chances of identifying and correcting them. We have mentioned some of the aspects of experimental design that may impact on both of these objectives. Utilization of bar codes from the earliest stages of sample preparation, and the automation of processing to as great an extent as possible would seem desirable to minimize the number of plating errors, or maximize the chances of detecting any that do occur.

The confirmation of some sample switches via STR genotyping is beneficial, but the level of BADGER evidence for some mix-ups is so compelling that the validation of all sample switches in this way would be a waste of time and resources. Moreover, there will not always be sufficient residual DNA in the sample plates to validate successfully in this manner, nor can all errors (particularly errors in transferring from plate to microarray) be identified with this approach. Given the considerable efforts required to conduct such validation, any additional methods that might provide evidence (e.g. video recording of all sample preparation) should be given consideration.

Our exploitation of cis-eQTL artefacts ignores one particular resource. Since the second generation of Illumina BeadArray, there have been large numbers of pairs of probes that differ at a single nucleotide (183 pairs in the fourth generation, 166 pairs in the third, 176 pairs in the second, and 9 pairs in the first). These pairs of probes most often differ at a known SNP, and so could provide a stronger association between genotype and expression measurements, although there are no guarantees that the particular genes involved will be useful for any particular study.

The BADGER approach to identifying errors can undoubtedly be extended and improved. We have already mentioned that more informative priors could be used. Also tissue-specific eQTLs could be used to identify errors where multiple tissues are being contributed from each participant. We have been anticipating that the process will begin with an intended matching of expression and SNP arrays, however if pre-defined eQTLs are used (either known tissue specific eQTLs or cis-eQTL artefacts) then it will be possible to infer the matching of arrays from scratch.

A similar approach can be adopted for studies involving other data types, for example methylation and miRNAs, as well as purely expression studies involving multiple tissues where we might want to predict genotypes from each tissue-type to validate the identities of samples. There may be other resources on the arrays that could be exploited to aid mapping. Ultimately though, what matters is not how we find and correct the errors, but that we can and do find and correct them.

## Methods

### Overview of Data Used

Across a number of studies we have assembled a large number of genomic measurements of breast cancer samples (>2,500 Affymetrix SNP 6 genotyping arrays and >3,000 Illumina HT12 v3.0 expression arrays. The vast majority of these are primary tumour samples, but some of them correspond to matched normal tissue. In this paper we focus on the largest subset of these, the 2,136 expression and 2,465 genotyping arrays published in the METABRIC study [14] (2122 of these are matched). Amongst these, for 127 tumours, a complete set of high-quality genotype and expression arrays for both tumour and matched normal tissues was identified, the tumour and normal genotype arrays being clearly matched. The data generated by METABRIC are revised regularly as annotations and clinical information become available, thus the numbers presented here may differ a little from those presented in the METABRIC paper [14].

All results presented pertain to the published METABRIC arrays except for Figures 2 and 4, which by their nature pertain to arrays that it would not be appropriate to include in a mature data set, and the initial derivation of the eQTL set that was defined across all of the breast cancer data available at the end of the METABRIC pilot study (some of which did not contribute directly to the METABRIC dataset). For access to the array data, one should see the METABRIC paper.

### Definition of Our eQTL Set

Rather than using an externally defined set, we define our initial set of eQTLs from the data, but using only the 96 samples that formed an internal pilot for the study. Due to the additional scrutiny to which the pilot was subjected, the low number of samples available at the time (thus limiting scope for errors), and the relatively ‘low-throughput’ nature of this stage of the study, we are confident that no samples were switched in this data set.

We look for cis-acting eQTLs (log_10_(*p*)>15 and the SNP within 1 megabase of the expression probe) and considered only the expression probes with perfect annotation. This last criterion is possibly excessive, but still returns 383 eQTLs for our data set, despite the relatively small number of arrays from which we are deriving them.

### Overview of BADGER Method

The method we have used for finding plating errors we have termed BADGER, ostensibly standing for “BeadArray Diagnostic for Genotype and Expression Relationships”, but also recognizing the ‘nagging’ characteristic of the iterative procedure. We begin with the intended mapping of samples to arrays, and a set of strongly associated pairs of expression and genotyping probes (eQTLs). Using our samples, these associations are quantified and then used to ‘predict’ genotypes corresponding to each expression array.

For every combination of expression and genotype array a distance (the BADGER score) is calculated between the predicted genotypes from the expression array and the observations from the genotyping array. For each expression array, the matches to all genotyping arrays are then compared and ranked such that the best match is of rank 1. If the genotyping array that was thought to match the expression array is of rank 1, then we accept this mapping. Otherwise we search to see whether there is a likely plating error. If a plating error is found then (ideally after validation) we adjust the annotation of the samples on the arrays accordingly and return to the beginning. At this step we may also refine our set of eQTLs, removing any that show little or no diagnostic value. We judge an eQTL pair of expression and SNP probes based on the squared differences between the observed call from the SNP probe and the predicted SNP from the expression probe, summed over all pairs of arrays that we believe to match well.

### Genotype Prediction

We consider only three possible genotypes (AA, AB and BB), despite the possible non-diploid status of the samples we are investigating. Under our diploid assumption we can represent these numerically by taking as our statistic the B-allele count (0,1, or 2 respectively). For a given eQTL pair of an expression probe and genotype (indexed by *i*), and a set of known well-mapped arrays, we use kernel density estimation techniques to generate three density functions for expression, corresponding to the three genotypes. These we denote 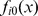, 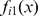, and 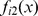 in the obvious manner.

For each expression array, *j*, we take the expression level associated with the 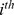 eQTL, 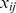, and estimate the value of the three density functions at that level (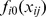, 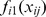, and 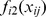). Giving each genotype an equal prior probability, we obtain posterior estimates for the B-allele count of


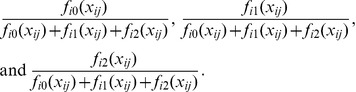
(1)

We then take as our predicted B-allele count the posterior expected value


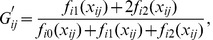
(2)

while our observed B-allele count for the probe associated with the 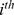 eQTL, on the 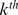 genotype array, we denote 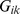.

### Comparison of Predicted and Observed B-allele Counts

Without loss of generality, assume we have *l* expression arrays and *m* genotype arrays where the first *n* of each type should correspond to the same *n* samples (i.e., 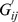 should be an estimate of 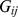 if 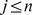 but is not expected to be if 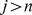). We construct an 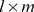 matrix of BADGER scores, **B**, where


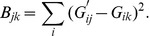
(3)

Naturally, the smaller the value of 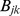, the better the match between the predicted and observed genotypes. Thus we can obtain the BADGER rank of the match (recalling that we are working from expression to genotype arrays), 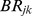, as the rank of 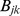 amongst the set 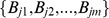. Note then that the diagonal of the 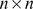 upper-left sub-matrix of **BR** will ideally be a vector of 1 s.

### Refinement of eQTL Set and Iteration

The performance of our eQTLs in the prediction of genotypes is not guaranteed, as this was not the criterion on which they were selected. Thus we choose to refine the set based on the performance of the eQTL amongst the arrays we believe to be matched. We can define, for the 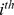 eQTL, a discrepancy statistic


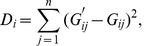
(4)

which allows us to identify a cut off 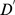 whereupon we will discard all eQTL pairs for which 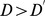. The value of 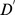 to use is somewhat arbitrary and can be suggested by consideration of the distribution of the 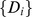. In our case, an inspection of a histogram of 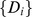 suggested that 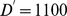 would be a suitable cutoff. This reduces our set of eQTLs from 383 to 125. Since our SNPs generally have a high minor allele frequency, and we anticipate few mismatches, a value of 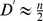 (as we have here with 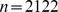) might not be unreasonable.

After refining our eQTL set, our BADGER scores can be quickly recalculated from the existing 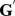 matrix.


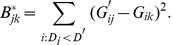
(5)

If we see evidence that there are mismatched arrays, then the process must return to step 1, as the eQTL densities will need to be recalculated. In this case, it seems prudent to reinstate the full eQTL set.

### Assessment of BADGER Performance

The set of BADGER scores associated with an expression array consists mainly of scores for non-matching arrays and just one (or a few, depending on the design) for a matching array. If our eQTL list is long enough, or our value of 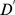 small enough, then we expect the BADGER score associated with the true match to be well-separated from the bulk of the BADGER scores.

Consequently, we sort the set 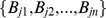 to generate order statistics 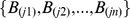 such that 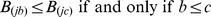. Ideally, 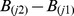 (the difference between the smallest and second smallest observations) will then be large. If the set of genotype arrays contains multiple arrays on which samples from the same person have been run, then this is not the difference at which to look. Thus the statistic.


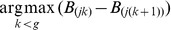
(6)

is required, where *g* is the largest number of replicate genotype arrays that we might encounter in the data set.

### Choice of Prior

Our choice of prior is somewhat naive; we might for example make better use of known allele frequencies. Alternatively, if desiring a ‘fair’ prior, we might wish to give more weighting to the AB genotype, however our use of the expected B-allele count ensures that as the information from the expression data decreases the predicted genotype already tends to the AB genotype. Thus we do not wish to punish too heavily differences in observed and predicted B-allele counts when the predicted count is 1. Nor do we wish to punish errors that could be genotype miscalling, which will nearly always be between heterozygous and homozygous rather than going from AA to BB.

### Metrics for Expression Array Quality

A convenient measure of signal quality for the Illumina expression BeadArray is the 95th percentile of log-intensities (P95) reported by the scanner. This is a general indication of the signal level, and while a useful quality statistic, it has no absolute interpretation as batch effects and scanner settings can cause large variation in the p95 value. This is true even after adjusting for the similarly defined P05 value which can be regarded as a baseline.

We consider a second metric for the quality of signal on an array. This statistic we denote 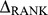 and we base on the re-annotation of the platform [22]. Here the average rank of the probes (high rank  =  high expression) that are considered to be well designed (denoted ‘perfect’ in the annotation files) is compared to the average rank of the probes that map to intergenic regions and so are not expected to show signal. While this statistic might vary between tissues, between sexes, and between conditions (and so require some adjustment to compensate for predictably different patterns of expression), within a consistent tissue type such as we have here it should be less affected by batch effects. For this reason we may risk interpreting it in an absolute manner. Unlike P95, the 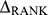 statistic is not generated automatically, and we only have its value for a large subset (including the METABRIC study arrays) of the data made available to us. For data obtained from public repositories, the 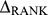 statistic may be as easy to calculate as the P95.

For an overview of Illumina expression array quality metrics, see Ritchie et al. [25].

### Detection of Plating Errors

In the majority of cases the genotype array that is meant to match the expression array will have a BADGER rank of 1. This genotype and expression array can then be removed from the analysis. The number of potential plating errors will hopefully be small enough that the manual assessment of each case will be feasible. Our experience is that the majority of simple cases can be resolved easily by considering neighbouring arrays as potential matches (either simple swaps, or accidental duplications). Care must be taken that one is identifying the entire error and not just a part of it. For example if the order of four consecutive arrays was reversed, then the middle two arrays might be identified as a simple pair of swapped neighbours and removed from further analysis, hindering the detection of the nature of the error for the outer two arrays.

If the design of the experiment allows, indeed if plating details are available for all stages, then we may be able to write code to investigate likely plating errors (use of the wrong plate, rotations of plate, reversal of BeadChip etc.) systematically, and report if any substantially increase the agreement between expression and genotype arrays. Else we can use graph theoretic techniques to suggest errors.

The problem can be represented as a bipartite graph where one set of nodes represents unresolved expression arrays and the other set of nodes represents unresolved genotyping arrays. An edge is then drawn between two nodes if the BADGER rank for that expression array/genotyping array combination is less than some threshold. Swaps will then appear as short cycles in the graph, more complicated rearrangements will appear either as long cycles (detectable e.g. using the Floyd-Warshall algorithm) or long chains on the graph. These can be detected algorithmically or spotted easily by eye if the graph is visualized.

### Validation of Plating Errors

Where BADGER suggested that there existed an inconsistency between the expression and genotype data, and the scale of the inconsistency is small enough that the BADGER prediction alone is not sufficient evidence, validation has proven possible via the residual DNA to be found in the wells of RNA extraction plates. This approach is, of course, dependent on enough DNA being present in those wells.

### Code Availability

Sweave S1 and Sweave S2 contain the documents and additional data required to reproduce the results presented here. In addition, a BADGER R package is being developed and maintained at http://badger.r-forge.r-project.org/.

## Supporting Information

Sweave S1 Sweave file [27] and supporting data to enable reproduction of the ‘Close relatives and validation’ section of this article.
(ZIP)

Sweave S2 Sweave file [27] and supporting data to enable reproduction of analyses not relating to the ‘Close relatives and validation’ section of this article.
(ZIP)
