# Supplementary material for: Calling Sample Mix-Ups in Cancer Population Studies
Source: PLoS One. 2012 Aug 9;7(8):e41815. doi: 10.1371/journal.pone.0041815 (PMC3415393; doi:10.1371/journal.pone.0041815)
Supplement: Sweave S1 — Sweave file [27] and supporting data to enable reproduction of the ‘Close relatives and validation’ section of this article. (ZIP) [file pone.0041815.s001.zip › HapMapSweave.pdf]

Code to reproduce HapMap/GeneVar results and figures in the paper "Calling sample mixups in cancer population studies"

Andy Lynch
Department of Oncology, University of Cambridge

1 Introduction and preparations

In this analysis we take the BADGER methods and apply them to one of the data sets considered in the MixupMapper paper. To achieve this, it is necessary to go to the MixupMapper page at `http://genenetwork.nl/wordpress/mixupmapper/`, head to the section entitled "Additional data to the manuscript" and download the files `Stranger.zip`, `Stranger-CHB+JPT.zip`, `Stranger-CEU.zip`, and `Stranger-YRI.zip`. For this document, this was done on 15/06/2012.

- From `Stranger.zip` extract the file `IlluminaExpressionDataOriginalWithDuplicates.txt`.
- From `Stranger-CEU.zip` extract the file `/eQTLMapping-Uncorrected/eQTLProbesFDR0.05.txt` and rename it `eQTLCEU.txt`.
- From `Stranger-CEU.zip` extract the file `/Mix-up-Analysis-Uncorrected/SuggestedCouplings.txt` and rename it `SuggCoupCEU.txt`.
- From `Stranger-CHB+JPT.zip` extract the file `/eQTLMapping-Uncorrected/eQTLProbesFDR0.05.txt` and rename it `eQTLCHBJPT.txt`.
- From `Stranger-CHB+JPT.zip` extract the file `/Mix-up-Analysis-Uncorrected/SuggestedCouplings.txt` and rename it `SuggCoupCHBJPT.txt`.
- From `Stranger-YRI.zip` extract the file `/eQTLMapping-Uncorrected/eQTLProbesFDR0.05.txt` and rename it `eQTLYRI.txt`.
- From `Stranger-YRI.zip` extract the file `/Mix-up-Analysis-Uncorrected/SuggestedCouplings.txt` and rename it `SuggCoupYRI.txt`.

Note the suggested couplings for YRI naturally don't include the mixed up case (NA18515), so this shall have to be added later.

2 Load required packages

```
> library(compiler)
```

3 Expression Matrices

We now use these files to generate the expression matrices that BADGER will consider. First load in the data

```
> FullEXP<-read.table("IlluminaExpressionDataOriginalWithDuplicates.txt",header=T,as.is=T)
```

Now note that there are some issues with one of the NA10856 arrays, which is apparently a duplicate of a NA18503 array. The GSM-indexed files on GEO are not replicates, so it is not clear why this happens. In any case, keep it in mind as it explains one of the mismatches that we see later.

```
> # evidence of problems in matrix
> colnames(FullEXP)[1814]
```

```
[1] "GSM232802_NA18503_1_1"
```

```
> FullEXP[1:10,1814]
```

```
 [1] -0.6594490 -0.2036760  1.9410100  0.3126170  3.1938900 -0.4600370
 [7] -0.6681270  1.6597700 -0.4290710 -0.0761479
```

```
> colnames(FullEXP)[1752]
```

```
[1] "GSM232786_NA10856_2_2"
```

```
> FullEXP[1:10,1752]
```

```
 [1] -0.6594490 -0.2036760  1.9410100  0.3126170  3.1938900 -0.4600370
 [7] -0.6681270  1.6597700 -0.4290710 -0.0761479
```

There are a bunch of other arrays that are duplicates, and we shall remove these now.

```
> ## remove duplicates
> temp<-FullEXP[,-(1:9)]
> tempcor<-(cor(temp[1:20,]))
> diag(tempcor)<-0
> FullEXP<-FullEXP[-(row(tempcor)[tempcor==1])[1:685]]
```

Now produce three expression matrices (one for CEU, one for YRI and one for CHB and JPT combined.)

```
> YRIeQTLs<-read.table("eQTLYRI.txt",header=T,as.is=T)
> YRIEXP<-FullEXP[match(YRIeQTLs$ProbeName,FullEXP[,1]),]
> YRIEXP<-YRIEXP[,-(1:9)]
> YRIEXP<-as.matrix(YRIEXP)
> YRIsamps<-read.table("SuggCoupYRI.txt",header=F,as.is=T)
> YRIEXPNames<-sapply(colnames(YRIEXP),function(x){unlist(strsplit(as.character(x),"_"))[2]})
> colnames(YRIEXP)<-YRIEXPNames
> YRIEXP<-YRIEXP[,YRIEXPNames %in% c("NA18515",YRIsamps[,1])]
> YRIEXPNames<-colnames(YRIEXP)
> rownames(YRIEXP)<-YRIeQTLs$ProbeName
> CEUeQTLs<-read.table("eQTLCEU.txt",header=T,as.is=T)
> CEUEXP<-FullEXP[match(CEUeQTLs$ProbeName,FullEXP[,1]),]
> CEUEXP<-CEUEXP[,-(1:9)]
> CEUEXP<-as.matrix(CEUEXP)
> CEUEXPNames<-sapply(colnames(CEUEXP),function(x){unlist(strsplit(as.character(x),"_"))[2]})
> colnames(CEUEXP)<-CEUEXPNames
> CEUsamps<-read.table("SuggCoupCEU.txt",header=F,as.is=T)
> CEUEXP<-CEUEXP[,CEUEXPNames %in% CEUsamps[,1]]
> CEUEXPNames<-colnames(CEUEXP)
> rownames(CEUEXP)<-CEUeQTLs$ProbeName
> CHBJPTeQTLs<-read.table("eQTLCHBJPT.txt",header=T,as.is=T)
> CHBJPTEXP<-FullEXP[match(CHBJPTeQTLs$ProbeName,FullEXP[,1]),]
> CHBJPTEXP<-CHBJPTEXP[,-(1:9)]
> CHBJPTEXP<-as.matrix(CHBJPTEXP)
> CHBJPTEXPNames<-sapply(colnames(CHBJPTEXP),function(x){unlist(strsplit(as.character(x),"_"))[2]})
> colnames(CHBJPTEXP)<-CHBJPTEXPNames
> CHBJPTsamps<-read.table("SuggCoupCHBJPT.txt",header=F,as.is=T)
> CHBJPTEXP<-CHBJPTEXP[,CHBJPTEXPNames %in% CHBJPTsamps[,1]]
> CHBJPTEXPNames<-colnames(CHBJPTEXP)
> rownames(CHBJPTEXP)<-CHBJPTeQTLs$ProbeName
```

4 SNP Matrices

The SNP matrices from Westra et al are in a bespoke format, and not easy with which to work. Instead we download the SNPs from the hapmap Biomart (This occurred on the 10th [YRI] and 17th of June [CEU, JPT, CHB]) at http://hapmap.ncbi.nlm.nih.gov/biomart/martview/. Note that there are individuals with many missing values, for which we shall have to make some adjustments. Also note that the minor allele frequency is quite low in many of the SNPs.

The list of eQTLs contains multiple entries for the same SNP on occasions. In a 'real' analysis we would recommend avoiding this. Similarly, we would recommend sticking to the autosomal chromosomes. However, for this illustration we proceed. When defining the eQTL relationships, one would also have to be careful to take into account the family structures.

```
> YRISNPs<-read.delim("YRISNPdata.txt",as.is=T)
> YRISNPMAT<-matrix(NA,nrow=634,ncol=176)
> colnames(YRISNPMAT)<-unlist(strsplit(YRISNPs[1,4]," "))
> for(i in 1:634){
+   YRISNPMAT[i,]<-unlist(strsplit(YRISNPs[i,5]," "))
+   }
> YRISNPMAT[YRISNPMAT=="NN"]<-NA
> for(i in 1:634){
+   YRISNPMAT[i,]<-as.numeric(as.factor(YRISNPMAT[i,]))
+   } #We have 29 SNPs used in two eQTL relationships
> YRISNPMAT<-YRISNPMAT[match(YRIeQTLs[,4],YRISNPs[,2]),]
> YRISNPMAT<-YRISNPMAT[,colnames(YRISNPMAT) %in% YRIEXPNames]
> for(i in 1:dim(YRISNPMAT)[1]){
+   for(j in 1:dim(YRISNPMAT)[2]){
+     YRISNPMAT[i,j]<-as.numeric(as.character(YRISNPMAT[i,j]))
+     }
+   }
> mode(YRISNPMAT)<-"numeric"
> CEUSNPs<-read.delim("CEUSNPdata.txt",as.is=T)
> CEUSNPMAT<-matrix(NA,nrow=875,ncol=174)
> colnames(CEUSNPMAT)<-unlist(strsplit(CEUSNPs[1,3]," "))
> for(i in 1:875){
+   CEUSNPMAT[i,]<-unlist(strsplit(CEUSNPs[i,4]," "))
+   }
> CEUSNPMAT[CEUSNPMAT=="NN"]<-NA
> for(i in 1:875){
+   CEUSNPMAT[i,]<-as.numeric(as.factor(CEUSNPMAT[i,]))
+   } #We have 28 SNPs used in twoeQTL relationships
> CEUSNPMAT<-CEUSNPMAT[match(CEUeQTLs[,4],CEUSNPs[,2]),]
> CEUSNPMAT<-CEUSNPMAT[,colnames(CEUSNPMAT) %in% CEUEXPNames]
> for(i in 1:dim(CEUSNPMAT)[1]){
+   for(j in 1:dim(CEUSNPMAT)[2]){
+     CEUSNPMAT[i,j]<-as.numeric(as.character(CEUSNPMAT[i,j]))
+     }
+   }
> mode(CEUSNPMAT)<-"numeric"
> CHBJPTSNPs<-read.delim("CHBJPTSNPdata.txt",as.is=T) # from hapmap biomart
> CHBJPTSNPMAT<-matrix(NA,nrow=1437,ncol=175)
> colnames(CHBJPTSNPMAT)<-c(unlist(strsplit(CHBJPTSNPs[1,3]," ")),unlist(strsplit(CHBJPTSNPs[2,3]," "))
> for(i in 1:1437){
+   CHBJPTSNPMAT[i,1:86]<-unlist(strsplit(CHBJPTSNPs[2*i-1,4]," "))
+   CHBJPTSNPMAT[i,87:175]<-unlist(strsplit(CHBJPTSNPs[2*i,4]," "))
+   }
```

```
> CHBJPTSNPMAT[CHBJPTSNPMAT=="NN"]<-NA
> for(i in 1:1437){
+   CHBJPTSNPMAT[i,]<-as.numeric(as.factor(CHBJPTSNPMAT[i,]))
+   } #We have 74 SNPs used in two eQTL relationships
> CHBJPTSNPMAT<-CHBJPTSNPMAT[match(CHBJPTeQTLs[,4],CHBJPTSNPs[seq(1,2873,2),2]),]
> CHBJPTSNPMAT<-CHBJPTSNPMAT[,colnames(CHBJPTSNPMAT) %in% CHBJPTEXPNames]
> for(i in 1:dim(CHBJPTSNPMAT)[1]){
+   for(j in 1:dim(CHBJPTSNPMAT)[2]){
+     CHBJPTSNPMAT[i,j]<-as.numeric(as.character(CHBJPTSNPMAT[i,j]))
+     }
+   }
> mode(CHBJPTSNPMAT)<-"numeric"
```

Rather than processing the files, we will just load in some we prepared earlier, along with family id information.

```
> load("SNPMatrices.rda")
```

5 BADGER functions

Once the BADGER package is available, it will be simpler to load that. Until then, we define the functions we wish to use.

```
> BADGERpredSNP<-function(MATEXP,MATSNP,BADGEREXP){
+ if(is.null(BADGEREXP)){BADGEREXP<-MATEXP}
+   preds<-matrix(NA,nrow=nrow(BADGEREXP),ncol=ncol(BADGEREXP))
+   for(i in 1:(nrow(MATEXP))){
+                 #cat(i," ")
+                 if(sum(MATSNP[i,]==1,na.rm=T)>2){dens1<-density((MATEXP)[i,which(MATSNP[i,]==1)],na.rm
+                 if(sum(MATSNP[i,]==2,na.rm=T)>2){dens2<-density((MATEXP)[i,which(MATSNP[i,]==2)],na.rm
+                 if(sum(MATSNP[i,]==3,na.rm=T)>2){dens3<-density((MATEXP)[i,which(MATSNP[i,]==3)],na.rm
+                 for(j in 1:(ncol(BADGEREXP))){
+                         useval<-BADGEREXP[i,j]
+                         if(is.na(useval)){useval<-mean(MATEXP[i,],na.rm=T)}
+                         pred1<-0
+                         pred2<-0
+                         pred3<-0
+                         if(sum(MATSNP[i,]==1,na.rm=T)>2){pred1<-dens1$y[which.min(abs(dens1$x-useval)
+                         if(sum(MATSNP[i,]==2,na.rm=T)>2){pred2<-dens2$y[which.min(abs(dens2$x-useval)
+                         if(sum(MATSNP[i,]==3,na.rm=T)>2){pred3<-dens3$y[which.min(abs(dens3$x-useval)
+                         predsum<-pred1+pred2+pred3
+                         pred1<-pred1/predsum
+                         pred2<-pred2/predsum
+                         pred3<-pred3/predsum
+                         preds[i,j]<-(pred1+2*pred2+3*pred3)
+                 }
+         }
+         colnames(preds)<-colnames(BADGEREXP)
+         return(preds)
+ }
> B.predSNP<-cmpfun(BADGERpredSNP)
> BADGERmatchScores<-function(predictedSNP,BADGERSNP,useEQTL=NULL){
+   if(is.null(useEQTL)){useEQTL<-1:(nrow(BADGERSNP))}
+         matchscore<-matrix(NA,nrow=ncol(predictedSNP),ncol=ncol(BADGERSNP))
```

```
+               for(pred in 1:ncol(predictedSNP)){
+                       for(obs in 1:ncol(BADGERSNP)){
+                               matchscore[pred,obs]<-sum((predictedSNP[useEQTL,pred]-
+                               as.numeric(BADGERSNP[useEQTL,obs]))^2,na.rm=T)
+                       }
+               }
+       return(matchscore)
+       }
> B.MS<-cmpfun(BADGERmatchScores)
> QTLres<-function(predictedMATSNP,MATSNP){
+   matchscore<-rep(NA,nrow(MATSNP))
+               for(EQTL in 1:nrow(MATSNP)){
+                       matchscore[EQTL]<-sum((predictedMATSNP[EQTL,]
+                            -as.numeric(MATSNP[EQTL,]))^2,na.rm=T)
+               }
+       return(matchscore)
+       }
> B.RES<-cmpfun(QTLres)
```

6 Perform basic BADGER analysis

For each population, we predict the genotype for each expression value.The sum of squared residual values shows a definite multi-modality, and 25 seems a fairly natural cutoff by which to filter the eQTLs to use in generating the BADGER score..

```
> YRIpredSNP<-B.predSNP(YRIEXP[,match(colnames(YRISNPMAT),YRIEXPNames)],YRISNPMAT,YRIEXP)
> CEUpredSNP<-B.predSNP(CEUEXP[,match(colnames(CEUSNPMAT),CEUEXPNames)],CEUSNPMAT,CEUEXP)
> CHBJPTpredSNP<-B.predSNP(CHBJPTEXP[,match(colnames(CHBJPTSNPMAT),CHBJPTEXPNames)],CHBJPTSNPMAT,
+ CHBJPTEXP)
> YRIeQTLres<-B.RES(YRIpredSNP[,match(colnames(YRISNPMAT),YRIEXPNames)],YRISNPMAT)
> CEUeQTLres<-B.RES(CEUpredSNP[,match(colnames(CEUSNPMAT),CEUEXPNames)],CEUSNPMAT)
> CHBJPTeQTLres<-B.RES(CHBJPTpredSNP[,match(colnames(CHBJPTSNPMAT),CHBJPTEXPNames)],CHBJPTSNPMAT)
> hist(YRIeQTLres,xlab="residual sum of squares for predicted and observed B-allele counts",ylab="number
> YRIMS<-B.MS(YRIpredSNP,YRISNPMAT,which(YRIeQTLres<25))
> CEUMS<-B.MS(CEUpredSNP,CEUSNPMAT,which(CEUeQTLres<25))
> CHBMS<-B.MS(CHBJPTpredSNP,CHBJPTSNPMAT,which(CHBJPTeQTLres<25))
```

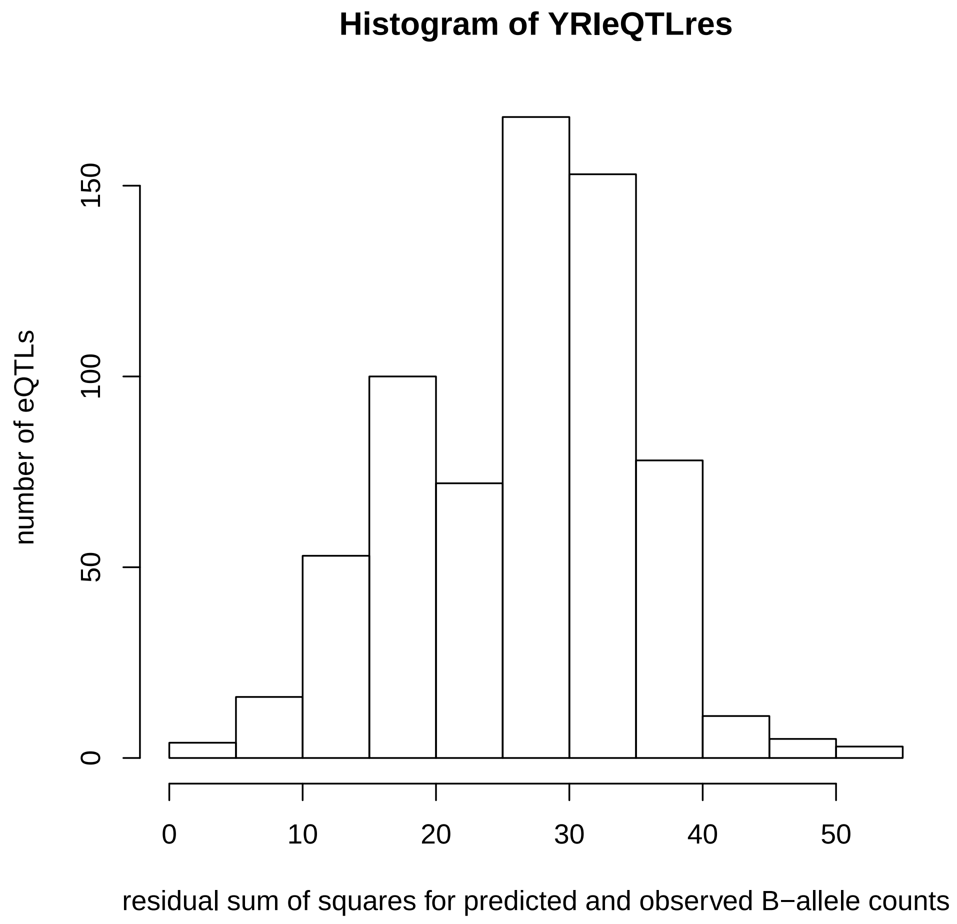

Now we get rid of the values associated with the samples that have virtually no SNP info, as these are unreliable. Then identify the problematic samples (those for which the sample ID in the expression array does not match the sample ID in the best matching genotype array).

```
> YRIMScens<-YRIMS
> YRIMScens[,which(apply(is.na(YRISNPMAT),2,sum)==658)]<-NA
> CEUMScens<-CEUMS
> CEUMScens[,which(apply(is.na(CEUSNPMAT),2,sum)>890)]<-NA
> CHBMScens<-CHBMS
> CHBMScens[,which(apply(is.na(CHBJPTSNPMAT),2,sum)>1000)]<-NA
> YRIMScheck<-rep(0,417)
> for(i in 1:417){
+ YRIMScheck[i]<-colnames(YRISNPMAT)[which.min(YRIMScens[i,])]
+ }
> YRIMScheck[YRIEXPNames %in% colnames(YRISNPMAT)[which(apply(is.na(YRISNPMAT),2,sum)==658)]]<-NA
> YRIEXPNames[which(YRIMScheck!=YRIEXPNames)]
```

```
[1] "NA18515" "NA18515" "NA18515" "NA18515"
```

```
> CEUMScheck<-rep(0,395)
> for(i in 1:395){
+ CEUMScheck[i]<-colnames(CEUSNPMAT)[which.min(CEUMScens[i,])]}
> CEUMScheck[CEUEXPNames %in% colnames(CEUSNPMAT)[which(apply(is.na(CEUSNPMAT),2,sum)>890)]]<-NA
> CEUEXPNames[which(CEUMScheck!=CEUEXPNames)]
```

```
[1] "NA10856"
```

```
> CHBMScheck<-rep(0,423)
> for(i in 1:423){
+ CHBMScheck[i]<-colnames(CHBJPTSNPMAT)[which.min(CHBMScens[i,])]}
> CHBMScheck[CHBJPTEXPNames %in%colnames(CHBJPTSNPMAT)[which(apply(is.na(CHBJPTSNPMAT),2,sum)>1400)]]<-
> CHBJPTEXPNames[which(CHBMScheck!=CHBJPTEXPNames)]
```

```
character(0)
```

7 Resolve the problem with the YRI sample

If we look at the genotype array that matches best to the expression arrays associated with NA18515, then we see that it is associated with NA18853 for all replicate expression arrays. This is also the case for the genotype arrays matching best to the expression arrays associated with NA18853.

When we look at the expression arrays matching best to the genotype array associated with NA18515, we see that the best matches are associated with NA18516 and NA18517 (the parents of NA18515). The matches are not great though. No more than one would expect for close relatives, so we can begin to suspect that the Genotype array is correct for NA18515, but that the expression arrays are actually NA18853 or a close relative. Indeed we can confirm that the genotype array for NA18515 is correctly annotated for sample, as the genotype comparison with the arrays for NA18516 and NA18517 confirm the relationship.

```
> par(mfrow=c(2,2))
> hist(YRIMScens[which(YRIEXPNames=="NA18515"),],breaks=seq(40,170,10),col="grey50",ylim=c(0,50),main=
+ "matching for NA18515 expression",xlab="SNP Array BADGER scores")
> hist(YRIMScens[which(YRIEXPNames=="NA18515"),c(10:12,17:18)],add=T,col="pink",breaks=seq(40,170,10))
> hist(YRIMScens[which(YRIEXPNames=="NA18515"),c(10:12,17)],add=T,col="red",breaks=seq(40,170,10))
> hist(YRIMScens[which(YRIEXPNames=="NA18515"),10:12],add=T,col="cyan",breaks=seq(40,170,10))
> hist(YRIMScens[which(YRIEXPNames=="NA18515"),11:12],add=T,col="darkblue",breaks=seq(40,170,10))
> hist(YRIMScens[which(YRIEXPNames=="NA18515"),12],add=T,col="blue",breaks=seq(40,170,10))
> legend(40,45,legend=c("NA18515","NA18516","NA18517","NA18853","NA18854"),fill=c("cyan","darkblue","bl
> hist(YRIMScens[which(YRIEXPNames=="NA18853"),],breaks=seq(40,170,10),col="grey50",ylim=c(0,50),main=
+ "matching for NA18853 expression",xlab="SNP Array BADGER scores")
> hist(YRIMScens[which(YRIEXPNames=="NA18853"),c(10:12,17:18)],add=T,col="pink",breaks=seq(40,170,10))
> hist(YRIMScens[which(YRIEXPNames=="NA18853"),c(10:12,17)],add=T,col="red",breaks=seq(40,170,10))
> hist(YRIMScens[which(YRIEXPNames=="NA18853"),10:12],add=T,col="cyan",breaks=seq(40,170,10))
> hist(YRIMScens[which(YRIEXPNames=="NA18853"),11:12],add=T,col="darkblue",breaks=seq(40,170,10))
> hist(YRIMScens[which(YRIEXPNames=="NA18853"),12],add=T,col="blue",breaks=seq(40,170,10))
> hist(YRIMScens[,10],breaks=seq(40,170,10),col="grey50",ylim=c(0,50),main="matching for NA18515
+ genotype",xlab="Expression Array BADGER scores")
> hist(YRIMScens[which(YRIEXPNames %in%
+ c("NA18515","NA18516","NA18517","NA18853","NA18854")),10],add=T,col="pink",breaks=seq(40,170,10))
> hist(YRIMScens[which(YRIEXPNames %in%
+ c("NA18515","NA18516","NA18517","NA18853")),10],add=T,col="red",breaks=seq(40,170,10))
> hist(YRIMScens[which(YRIEXPNames %in%
+ c("NA18515","NA18516","NA18517")),10],add=T,col="cyan",breaks=seq(40,170,10))
> hist(YRIMScens[which(YRIEXPNames %in%
+ c("NA18516","NA18517")),10],add=T,col="darkblue",breaks=seq(40,170,10))
> hist(YRIMScens[which(YRIEXPNames %in% c("NA18517")),10],add=T,col="blue",breaks=seq(40,170,10))
> hist(YRIMScens[,17],breaks=seq(40,170,10),col="grey50",ylim=c(0,50),main="matching for NA18853
+ genotype",xlab="Expression Array BADGER scores")
> hist(YRIMScens[which(YRIEXPNames %in%
+ c("NA18515","NA18516","NA18517","NA18853","NA18854")),17],add=T,col="pink",breaks=seq(40,170,10))
> hist(YRIMScens[which(YRIEXPNames %in%
```

```
+ c("NA18515","NA18516","NA18517","NA18853")),17],add=T,col="red",breaks=seq(40,170,10))
> hist(YRIMScens[which(YRIEXPNames %in%
+ c("NA18515","NA18516","NA18517")),17],add=T,col="cyan",breaks=seq(40,170,10))
> hist(YRIMScens[which(YRIEXPNames %in%
+ c("NA18516","NA18517")),17],add=T,col="darkblue",breaks=seq(40,170,10))
> hist(YRIMScens[which(YRIEXPNames %in% c("NA18517")),17],add=T,col="blue",breaks=seq(40,170,10))
```

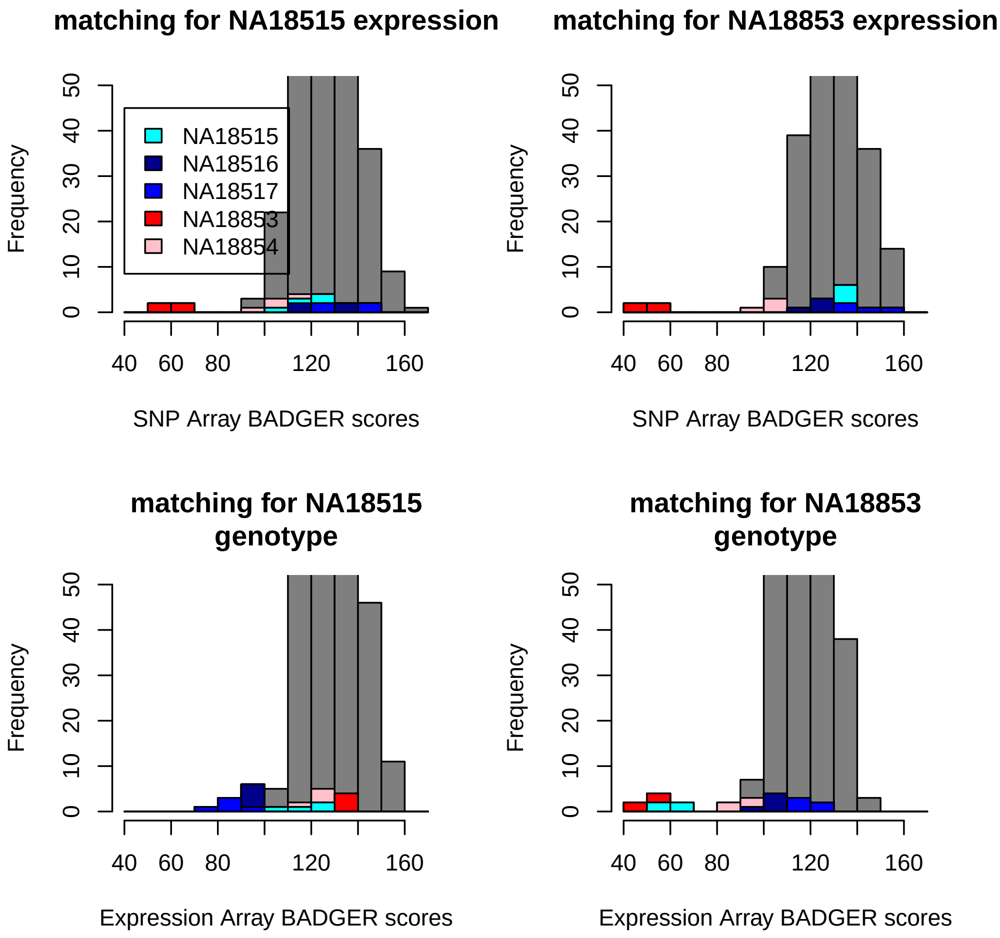

8 Close relatives, and their ability to throw BADGER.

While, on average, the match to a close relative is not as good as is the match to the correct ID, it is the case that a) the range of scores associated with close relatives encompasses the range seen for the correct sample IDs and b) that in the absence of a correct sample, the relative may appear to be an outlyingly good match and lead to a mismatch.

```
> par(mfrow=c(2,1))
> CEUmatchlevel<-matrix(0,nrow=395,ncol=90)
> for(f in 1:90){
+ tempnames1<-colnames(CEUSNPMAT)[f]
+ parpeds<-HMFAMFull[match(tempnames1,HMFAMFull[,1]),4:5]
+ if(!all(parpeds==0)){
+ tempfn<-HMFAMFull[match(tempnames1,HMFAMFull[,1]),2]
```

```
+ tempuse<-which((HMFAMFull[,2]==tempfn)&(HMFAMFull[,3] %in% parpeds))
+ tempnames2<-c(HMFAMFull[tempuse,1],tempnames1)
+ CEUmatchlevel[(CEUEXPNames %in% tempnames2),colnames(CEUSNPMAT) %in% tempnames2]<-1
+ }
+ CEUmatchlevel[(CEUEXPNames %in% tempnames1),colnames(CEUSNPMAT) %in% tempnames1]<-2
+ }
> CEUmatchlevel[338,]<-NA
> boxplot(as.vector(CEUMScens)~as.vector(CEUmatchlevel),names=c("unrelated","close\nrelative","correct\n
> YRImatchlevel<-matrix(0,nrow=417,ncol=90)
> for(f in 1:90){
+ tempnames1<-colnames(YRISNPMAT)[f]
+ parpeds<-HMFAMFull[match(tempnames1,HMFAMFull[,1]),4:5]
+ if(!all(parpeds==0)){
+ tempfn<-HMFAMFull[match(tempnames1,HMFAMFull[,1]),2]
+ tempuse<-which((HMFAMFull[,2]==tempfn)&(HMFAMFull[,3] %in% parpeds))
+ tempnames2<-c(HMFAMFull[tempuse,1],tempnames1)
+ YRImatchlevel[(YRIEXPNames %in% tempnames2),colnames(YRISNPMAT) %in% tempnames2]<-1
+ }
+ YRImatchlevel[(YRIEXPNames %in% tempnames1),colnames(YRISNPMAT) %in% tempnames1]<-2
+ }
> YRImatchlevel[310:313,]<-NA
> boxplot(as.vector(YRIMScens)~as.vector(YRImatchlevel),names=c("unrelated","close\nrelative","correct\n
```

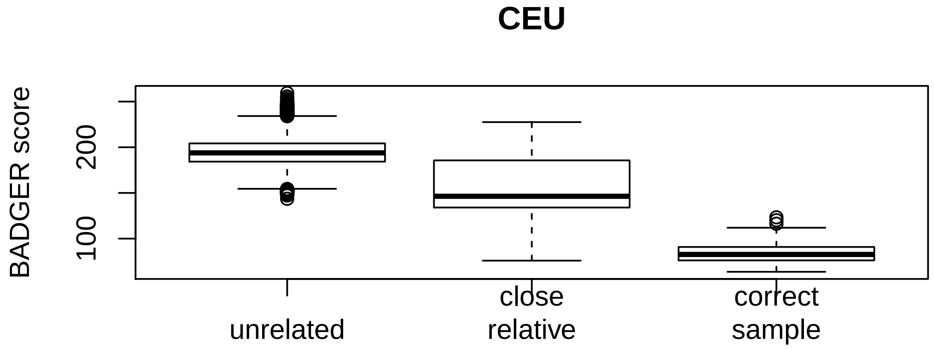

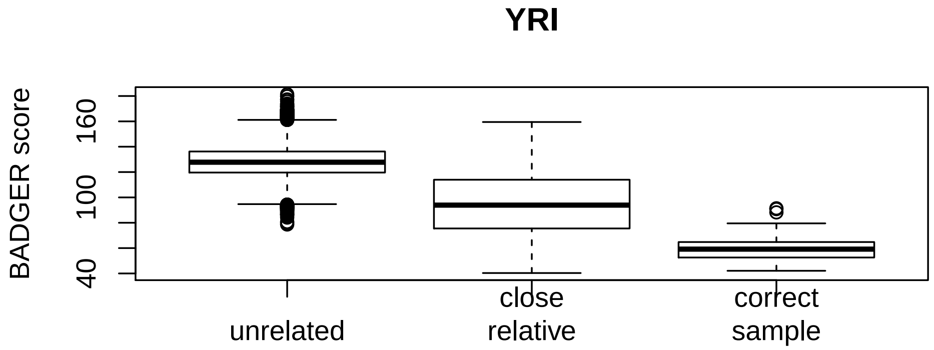

9 Simulation Exercise.

We run a series of simulations for the CHB+JPT populations. In each of these, a number of sample IDs will be switched in the genotype matrix, and BADGER will attempt to resolve them. The eQTLs we are considering were defined from the data set by Westra et al., so this is a bit of a cheat, but in contrast, and for the purposes of automation, the methods for calling switches in the population are primative.

```
> set.seed(23111963)
> myunrescount<-matrix(ncol=5,nrow=20)
> myresoutput<-matrix(ncol=5,nrow=20)
> for(indexi in c(1:20)){
+ # for full simulation, replace with ``for(indexi in 1:20){''
+   for(indexj in 1:5){
+ # for full simulation, replace with ``for(indexj in 1:5){''
+ itercount<-0
+ cat(indexi,"\t",indexj,"\n")
+ mixupnum<-indexi*4
+ mixups<-sample(which((apply(is.na(CHBJPTSNPMAT),2,sum)<1400)),mixupnum)
+
+
+ resolved<-F
+ needsiter<-T
+
+ simSNPMAT<-CHBJPTSNPMAT
+ for(i in 1:(mixupnum/2)){
+ simSNPMAT[,mixups[2*i-1]]<-CHBJPTSNPMAT[,mixups[2*i]]
+ simSNPMAT[,mixups[2*i]]<-CHBJPTSNPMAT[,mixups[2*i-1]]
+ }
+
+ colnames(simSNPMAT)<-colnames(CHBJPTSNPMAT)
+
+ while(needsiter){
+
+ cat(itercount,"\t")
+ cat(sum(apply(CHBJPTSNPMAT==simSNPMAT,2,all,na.rm=T)),"\t")
+ itercount<-itercount+1
+ simpredSNP<-B.predSNP(CHBJPTEXP[,match(colnames(simSNPMAT),CHBJPTEXPNames)],simSNPMAT,
+ CHBJPTEXP)
+ simeQTLres<-B.RES(simpredSNP[,match(colnames(simSNPMAT),CHBJPTEXPNames)],simSNPMAT)
+
+ #reslim<-max(sort(simeQTLres)[201],30)
+
+ simMS<-B.MS(simpredSNP,simSNPMAT,which(simeQTLres<35))
+ simMScens<-simMS
+ simMScens[,which(apply(is.na(simSNPMAT),2,sum)>1000)]<-NA
+
+ simMScheck<-rep(0,423)
+ for(i in 1:423){
+ simMScheck[i]<-colnames(simSNPMAT)[which.min(simMScens[i,])]}
+ simMScheck[CHBJPTEXPNames %in% colnames(simSNPMAT)[which(apply(is.na(simSNPMAT),2,sum)>1400)]]<-NA
+
+ #table(simMScheck[which(simMScheck!=CHBEXPNames)],CHBEXPNames[which(simMScheck!=CHBEXPNames)])
+
+ conslist<-unique(c(simMScheck[which(simMScheck!=CHBJPTEXPNames)],CHBJPTEXPNames[which(simMScheck!=CHB
+ stomplist<-NULL
```

```
+ temp<-simSNPMAT
+ madeswitch<-F
+ for(i in conslist){
+   potswitch<-unique(c(CHBJPTEXPNames[which(simMScheck==i)],simMScheck[which(CHBJPTEXPNames==i)]))
+         if(!(i %in% stomplist)){
+         if(length(potswitch)==1){
+         if(!(potswitch %in% stomplist)){
+                 if(potswitch!=i){
+                         simSNPMAT[,match(i,colnames(temp))]<-temp[,match(potswitch,colnames(temp))]
+                         simSNPMAT[,match(potswitch,colnames(temp))]<-temp[,match(i,colnames(temp))]
+                         #cat(i,potswitch,"\n")
+                         madeswitch<-T
+                         stomplist<-c(stomplist,i,potswitch)
+                 }
+         }}}
+ }
+ cat(stomplist,"\n")
+ colnames(simSNPMAT)<-colnames(CHBJPTSNPMAT)
+ if(!madeswitch){needsiter<-F}
+ if(itercount>30){needsiter<-F}
+ if(all(simSNPMAT==CHBJPTSNPMAT,na.rm=T)){
+ resolved=T}
+ #cat("madeswitch",madeswitch,"\n")
+ #cat("needsiter",needsiter,"\n")
+ }
+
+ myresoutput[indexi,indexj]<-itercount
+ myunrescount[indexi,indexj]<-90-sum(apply(CHBJPTSNPMAT==simSNPMAT,2,all,na.rm=T))
+ }}

1         1
0        86         NA18563 NA18545 NA18998 NA18608
1        90
1         2
0        86         NA18942 NA18542 NA18965 NA18637
1        90
1         3
0        86         NA18943 NA18550 NA18976 NA18624
1        90
1         4
0        86         NA18621 NA18603 NA18951 NA18633
1        90
1         5
0        86         NA18633 NA18623 NA18970 NA18943
1        90
2         1
0        82         NA18592 NA18550 NA18994 NA18605 NA18637 NA18623 NA18999 NA18940
1        90
2         2
0        82         NA18976 NA18550 NA18636 NA18566 NA18966 NA18611 NA18997 NA18990
1        90
2         3
0        82         NA18564 NA18542 NA18968 NA18571 NA18620 NA18579 NA18974 NA18961
1        90
```

2	4	
0	82	NA18579 NA18545 NA18997 NA18635 NA19005 NA18940 NA18970 NA18965
1	90	
2	5	
0	82	NA18577 NA18524 NA18995 NA18550 NA18967 NA18608 NA18945 NA18944
1	90	
3	1	
0	78	NA18943 NA18542 NA18576 NA18572 NA18973 NA18573 NA18965 NA18594 NA18972 NA18948 NA1
1	90	
3	2	
0	78	NA18608 NA18555 NA18995 NA18562 NA18978 NA18563 NA18969 NA18582 NA18999 NA18966 NA1
1	90	
3	3	
0	78	NA18620 NA18561 NA19000 NA18572 NA19005 NA18623 NA18981 NA18633 NA18987 NA18973 NA1
1	90	
3	4	
0	78	NA18960 NA18532 NA18593 NA18537 NA19007 NA18542 NA18570 NA18545 NA18964 NA18561 NA1
1	90	
3	5	
0	78	NA18592 NA18550 NA18966 NA18563 NA18987 NA18594 NA18975 NA18635 NA18940 NA18636 NA1
1	90	
4	1	
0	74	NA18965 NA18542 NA18994 NA18570 NA18978 NA18572 NA18967 NA18576 NA18594 NA18582 NA1
1	90	
4	2	
0	74	NA19007 NA18532 NA18573 NA18545 NA18605 NA18570 NA18624 NA18572 NA18999 NA18576 NA1
1	90	
4	3	
0	74	NA18956 NA18562 NA18959 NA18582 NA18995 NA18594 NA19000 NA18624 NA18969 NA18632 NA1
1	90	
4	4	
0	74	NA18563 NA18558 NA18944 NA18582 NA18608 NA18594 NA18987 NA18611 NA18952 NA18612 NA1
1	90	
4	5	
0	74	NA18965 NA18529 NA18969 NA18566 NA18980 NA18572 NA18637 NA18624 NA18960 NA18945 NA1
1	90	
5	1	
0	70	NA18636 NA18524 NA18608 NA18526 NA18632 NA18571 NA18573 NA18572 NA18997 NA18579 NA1
1	90	
5	2	
0	70	NA18620 NA18550 NA18960 NA18562 NA18940 NA18566 NA18976 NA18570 NA18966 NA18582 NA1
1	90	
5	3	
0	70	NA18622 NA18524 NA18974 NA18537 NA18944 NA18552 NA18990 NA18558 NA18633 NA18561 NA1
1	90	
5	4	
0	70	NA18998 NA18524 NA18995 NA18529 NA18971 NA18564 NA18605 NA18572 NA18987 NA18603 NA1
1	90	
5	5	
0	70	NA18956 NA18526 NA18592 NA18532 NA18605 NA18572 NA18942 NA18573 NA18636 NA18622 NA1
1	90	
6	1	
0	66	NA18611 NA18524 NA18632 NA18537 NA18951 NA18552 NA18972 NA18592 NA18980 NA18593 NA1
1	90	

6	2	
0	66	NA18562 NA18552 NA18612 NA18558 NA18999 NA18563 NA18943 NA18564 NA18961 NA18571 NA1
1	90	
6	3	
0	66	NA18563 NA18526 NA18572 NA18562 NA18964 NA18566 NA18605 NA18571 NA19000 NA18608 NA1
1	90	
6	4	
0	66	NA18622 NA18529 NA18940 NA18555 NA18592 NA18562 NA18632 NA18563 NA18576 NA18564 NA1
1	90	
6	5	
0	66	NA18980 NA18524 NA18555 NA18537 NA18949 NA18545 NA18576 NA18550 NA18974 NA18561 NA1
1	90	
7	1	
0	62	NA18970 NA18529 NA18608 NA18542 NA18563 NA18558 NA18945 NA18561 NA18991 NA18562 NA1
1	90	
7	2	
0	62	NA19000 NA18537 NA18545 NA18542 NA18555 NA18550 NA18636 NA18558 NA18621 NA18563 NA1
1	90	
7	3	
0	62	NA19005 NA18524 NA18532 NA18526 NA18947 NA18529 NA18605 NA18542 NA18970 NA18550 NA1
1	90	
7	4	
0	62	NA18633 NA18524 NA18995 NA18552 NA18980 NA18564 NA18945 NA18570 NA18635 NA18576 NA1
1	90	
7	5	
0	62	NA18947 NA18550 NA18945 NA18555 NA18980 NA18561 NA18573 NA18562 NA18611 NA18571 NA1
1	90	
8	1	
0	58	NA18582 NA18529 NA18997 NA18532 NA18621 NA18537 NA18978 NA18542 NA18980 NA18563 NA1
1	90	
8	2	
0	58	NA18545 NA18542 NA18959 NA18552 NA18605 NA18566 NA18969 NA18572 NA18974 NA18573 NA1
1	90	
8	3	
0	58	NA18577 NA18524 NA18603 NA18542 NA18994 NA18545 NA18960 NA18558 NA18949 NA18563 NA1
1	90	
8	4	
0	58	NA18975 NA18532 NA18561 NA18545 NA18573 NA18563 NA18967 NA18564 NA18624 NA18571 NA1
1	90	
8	5	
0	58	NA18980 NA18529 NA18960 NA18532 NA18942 NA18537 NA18572 NA18550 NA18999 NA18552 NA1
1	90	
9	1	
0	54	NA18959 NA18526 NA18621 NA18532 NA18951 NA18542 NA18608 NA18552 NA18594 NA18563 NA1
1	90	
9	2	
0	54	NA18951 NA18526 NA18997 NA18529 NA18605 NA18555 NA18961 NA18558 NA18577 NA18570 NA1
1	90	
9	3	
0	54	NA18965 NA18529 NA18571 NA18542 NA18594 NA18545 NA18566 NA18555 NA18960 NA18558 NA1
1	90	
9	4	
0	54	NA18995 NA18526 NA18978 NA18545 NA18633 NA18550 NA18592 NA18555 NA18566 NA18558 NA1
1	90	

9	5	
0	54	NA18608 NA18526 NA18635 NA18532 NA18942 NA18537 NA18990 NA18555 NA18637 NA18562 NA1
1	90	
10	1	
0	50	NA18537 NA18532 NA18975 NA18550 NA18966 NA18555 NA18637 NA18563 NA18945 NA18564 NA1
1	90	
10	2	
0	50	NA18972 NA18537 NA18943 NA18542 NA18944 NA18545 NA18995 NA18550 NA18974 NA18552 NA1
1	90	
10	3	
0	50	NA18959 NA18529 NA18550 NA18532 NA18570 NA18537 NA18571 NA18555 NA18999 NA18561 NA1
1	90	
10	4	
0	50	NA18593 NA18524 NA18561 NA18529 NA18947 NA18537 NA18635 NA18542 NA18966 NA18555 NA1
1	90	
10	5	
0	50	NA18961 NA18526 NA18972 NA18537 NA18945 NA18545 NA18592 NA18552 NA18609 NA18555 NA1
1	90	
11	1	
0	46	NA18636 NA18526 NA18603 NA18532 NA18972 NA18537 NA18969 NA18542 NA18571 NA18545 NA1
1	90	
11	2	
0	46	NA18623 NA18532 NA18960 NA18537 NA19007 NA18542 NA18563 NA18545 NA18612 NA18550 NA1
1	90	
11	3	
0	46	NA18524 NA18529 NA18964 NA18545 NA18967 NA18550 NA18970 NA18552 NA18577 NA18555 NA1
1	88	NA18997 NA18960
2	90	
11	4	
0	46	NA18621 NA18524 NA18940 NA18529 NA18561 NA18532 NA18612 NA18562 NA18573 NA18563 NA1
1	90	
11	5	
0	46	NA18959 NA18526 NA18555 NA18529 NA18609 NA18537 NA18972 NA18542 NA18635 NA18561 NA1
1	88	NA18623 NA18576
2	90	
12	1	
0	42	NA18532 NA18526 NA18967 NA18552 NA18952 NA18558 NA18945 NA18561 NA18577 NA18562 NA1
1	88	NA18975 NA18529
2	90	
12	2	
0	42	NA18605 NA18524 NA18632 NA18529 NA18624 NA18542 NA18991 NA18555 NA18943 NA18561 NA1
1	88	NA18981 NA18558
2	90	
12	3	
0	42	NA18532 NA18529 NA18632 NA18550 NA18573 NA18558 NA18608 NA18561 NA18637 NA18562 NA1
1	88	NA18956 NA18524
2	90	
12	4	
0	42	NA18951 NA18542 NA18566 NA18550 NA18605 NA18562 NA18633 NA18570 NA18970 NA18571 NA1
1	88	NA18635 NA18577
2	90	
12	5	
0	42	NA18976 NA18524 NA18940 NA18529 NA18608 NA18532 NA18633 NA18550 NA18956 NA18552 NA1
1	90	

13	1	
0	38	NA18555 NA18524 NA18594 NA18545 NA18959 NA18558 NA18972 NA18561 NA18636 NA18566 NA1
1	84	NA18995 NA18635 NA18980 NA18978 NA18999 NA18990
2	90	
13	2	
0	38	NA18995 NA18526 NA18999 NA18545 NA18964 NA18561 NA18972 NA18562 NA18633 NA18566 NA1
1	84	NA18608 NA18552 NA18966 NA18555 NA18991 NA18609
2	90	
13	3	
0	38	NA18991 NA18542 NA18980 NA18561 NA18635 NA18562 NA18995 NA18564 NA18592 NA18566 NA1
1	80	NA18944 NA18524 NA18952 NA18526 NA18582 NA18529 NA18997 NA18555 NA19007 NA18593
2	90	
13	4	
0	38	NA18572 NA18542 NA18552 NA18545 NA18620 NA18550 NA18980 NA18573 NA18943 NA18577 NA1
1	86	NA18562 NA18524 NA19000 NA18529
2	90	
13	5	
0	38	NA18636 NA18524 NA18997 NA18537 NA18998 NA18542 NA18635 NA18552 NA18940 NA18558 NA1
1	88	NA18952 NA18550
2	90	
14	1	
0	34	NA18973 NA18532 NA18959 NA18558 NA18967 NA18576 NA18951 NA18579 NA18594 NA18592 NA1
1	74	NA18571 NA18545 NA18998 NA18555 NA18975 NA18573 NA18981 NA18621 NA18966 NA18622 NA1
2	90	
14	2	
0	34	NA18978 NA18526 NA18632 NA18537 NA18945 NA18555 NA18608 NA18563 NA18564 NA18566 NA1
1	74	NA18558 NA18529 NA18956 NA18550 NA18999 NA18571 NA19000 NA18572 NA18623 NA18593 NA1
2	90	
14	3	
0	34	NA18969 NA18526 NA18973 NA18582 NA18632 NA18605 NA18624 NA18608 NA18592 NA18943 NA1
1	70	NA18609 NA18550 NA18636 NA18552 NA18971 NA18558 NA18965 NA18564 NA18949 NA18570 NA1
2	90	
14	4	
0	34	NA18945 NA18524 NA18542 NA18526 NA18582 NA18545 NA18637 NA18555 NA18611 NA18558 NA1
1	72	NA18550 NA18529 NA18959 NA18561 NA18990 NA18564 NA18968 NA18594 NA18966 NA18608 NA1
2	90	
14	5	
0	34	NA18981 NA18529 NA18577 NA18561 NA19005 NA18564 NA18972 NA18570 NA18633 NA18592 NA1
1	80	NA18566 NA18542 NA18576 NA18562 NA18605 NA18603 NA18997 NA18637 NA18966 NA18956
2	90	
15	1	
0	30	NA18980 NA18542 NA18974 NA18555 NA18987 NA18570 NA18970 NA18943 NA18940 NA18961 NA1
1	54	NA18966 NA18526 NA18576 NA18537 NA18947 NA18545 NA18964 NA18550 NA18594 NA18552 NA1
2	90	
15	2	
0	30	NA18577 NA18942 NA18974 NA18948 NA18637 NA18949 NA18573 NA18991 NA18545 NA18571 NA1
1	56	NA18603 NA18524 NA18947 NA18529 NA18635 NA18532 NA18609 NA18550 NA18975 NA18561 NA1
2	90	
15	3	
0	30	NA18964 NA18532 NA18987 NA18537 NA18981 NA18545 NA18975 NA18555 NA18967 NA18558 NA1
1	57	NA18995 NA18524 NA18633 NA18542 NA18632 NA18561 NA18593 NA18562 NA18945 NA18566 NA1
2	87	
15	4	
0	30	NA18526 NA18524 NA18612 NA18542 NA18978 NA18555 NA18968 NA18564 NA18973 NA18572 NA1

1	54	NA18620 NA18532 NA18570 NA18550 NA18961 NA18562 NA18975 NA18571 NA18990 NA18579 [illegible]
2	90	
15	5	
0	30	NA18532 NA18558 NA18969 NA18561 NA18545 NA18571 NA18582 NA18611 NA18995 NA18612 [illegible]
1	52	NA18975 NA18524 NA18633 NA18529 NA18999 NA18542 NA18972 NA18550 NA18964 NA18555 [illegible]
2	86	
16	1	
0	26	NA18592 NA18564 NA18558 NA18570 NA18994 NA18582 NA18620 NA18637 NA18949 NA19000 [illegible]
1	52	NA18965 NA18524 NA18623 NA18529 NA18635 NA18537 NA18964 NA18542 NA18980 NA18545 [illegible]
2	90	
16	2	
0	26	NA18959 NA18526 NA18635 NA18571 NA18940 NA18573 NA18951 NA18620 NA18969 NA18948 [illegible]
1	50	NA18563 NA18529 NA18624 NA18532 NA18990 NA18552 NA18994 NA18558 NA18524 NA18562 [illegible]
2	90	
16	3	
0	26	NA18974 NA18545 NA18942 NA18637 NA18967 NA18972 NA18582 NA18635 NA18612 NA18537 [illegible]
1	35	NA18555 NA18526 NA18572 NA18562 NA18965 NA18564 NA18953 NA18570 NA18995 NA18592 [illegible]
2	76	NA19005 NA18529 NA18981 NA18552 NA18956 NA18571 NA18622 NA18582 NA18980 NA18636 [illegible]
3	90	
16	4	
0	26	NA18612 NA18563 NA18636 NA18592 NA18971 NA18951 NA18573 NA18961 NA18633 NA18965 [illegible]
1	50	NA18593 NA18529 NA18609 NA18537 NA18959 NA18542 NA18944 NA18555 NA18623 NA18562 [illegible]
2	90	
16	5	
0	26	NA18635 NA18637 NA18636 NA18981 NA18959 NA18605 NA18970 NA18609 NA18971 NA18973
1	36	NA18572 NA18537 NA18592 NA18550 NA19000 NA18552 NA18542 NA18555 NA18952 NA18558 [illegible]
2	84	NA18980 NA18545 NA18947 NA18564 NA18972 NA18960
3	90	
17	1	
0	22	NA18636 NA18577 NA18965 NA18978 NA18550 NA18537
1	25	NA18561 NA18550 NA18564 NA18545
2	25	NA18537 NA18564 NA18570 NA18561 NA18942 NA18542 NA18967 NA18966 NA19000 NA18612
3	31	NA18997 NA18558 NA18945 NA18562 NA18959 NA18572 NA18972 NA18592 NA18573 NA18605 [illegible]
4	53	NA18593 NA18524 NA18621 NA18526 NA18620 NA18552 NA18998 NA18555 NA18637 NA18571 [illegible]
5	81	
17	2	
0	22	NA18542 NA19000 NA18563 NA18566 NA18572 NA18990 NA18573 NA18964 NA18967 NA18959
1	28	NA18997 NA18526 NA18636 NA18571 NA18975 NA18966 NA18987 NA18995 NA18537 NA18561 [illegible]
2	40	NA18550 NA18524 NA18969 NA18532 NA18633 NA18545 NA18974 NA18555 NA18564 NA18562 [illegible]
3	81	
17	3	
0	22	NA18965 NA18555 NA18967 NA18564 NA18552 NA18999 NA18563 NA18981 NA18570 NA18526 [illegible]
1	40	NA18970 NA18524 NA18579 NA18529 NA18636 NA18532 NA18573 NA18537 NA18998 NA18542 [illegible]
2	82	NA18612 NA18605 NA18940 NA18621
3	86	
17	4	
0	22	NA18995 NA18526 NA18558 NA18612 NA18573 NA18550 NA18577 NA18611 NA18594 NA18622 [illegible]
1	31	NA18592 NA18561 NA18971 NA18570 NA18940 NA18572 NA18545 NA18579 NA18532 NA18593 [illegible]
2	61	NA18964 NA18524 NA18968 NA18564 NA18961 NA18566 NA18972 NA18576 NA18965 NA18603 [illegible]
3	87	
17	5	
0	22	NA18995 NA18564 NA18624 NA18952 NA18537 NA18563
1	26	NA18960 NA18537 NA18980 NA18978 NA18994 NA18973
2	30	NA18971 NA18542 NA18552 NA18545 NA18987 NA18573 NA18577 NA18612 NA18967 NA18942 [illegible]

3	54	NA19007 NA18524 NA18594 NA18526 NA18611 NA18532 NA19000 NA18550 NA18999 NA18558 [illegible]
4	80	
18	1	
0	18	NA18545 NA18965 NA18577 NA19007 NA18612 NA18980
1	19	NA18526 NA18545 NA18611 NA18622 NA18636 NA18976
2	21	NA18971 NA18612 NA18972 NA18577 NA18975 NA18526
3	20	NA18529 NA18975 NA18577 NA18972 NA18635 NA18611 NA18942 NA18576
4	23	NA18532 NA18990 NA18573 NA18973 NA18969 NA18964
5	29	NA18624 NA18605 NA18999 NA18943 NA18995 NA18952 NA18563 NA18968 NA18564 NA18623 [illegible]
6	41	NA18633 NA18524 NA18555 NA18537 NA18994 NA18542 NA18947 NA18570 NA18991 NA18571 [illegible]
7	70	NA18621 NA18550 NA18981 NA18966
8	74	
18	2	
0	18	NA18532 NA18563 NA18542 NA18608 NA18633 NA18555 NA18995 NA18562 NA19000 NA18999
1	24	NA18537 NA18942 NA18571 NA18542 NA18949 NA18981 NA18956 NA18994 NA18980 NA18987 [illegible]
2	31	NA18526 NA18545 NA18524 NA18953 NA18577 NA18571 NA18967 NA18550 NA18969 NA18621 [illegible]
3	41	NA18975 NA18552 NA18961 NA18561 NA18576 NA18570 NA18608 NA18571 NA18579 NA18593 [illegible]
4	77	NA18976 NA18948
5	79	
18	3	
0	18	NA18635 NA18570 NA18577 NA18956 NA18949 NA18951 NA18952 NA18973
1	22	NA18953 NA18952 NA18972 NA19007
2	22	NA18978 NA18997
3	24	NA18987 NA18566 NA18974 NA18969
4	28	NA18545 NA18968 NA18558 NA18943 NA18609 NA18577 NA18970 NA19005 NA18994 NA18593 [illegible]
5	36	NA18542 NA18555 NA18576 NA18579 NA18632 NA18594 NA18999 NA18605 NA18991 NA18620 [illegible]
6	64	NA18940 NA18526 NA18980 NA18532 NA18621 NA18550 NA18611 NA18582 NA18966 NA18603 [illegible]
7	76	
18	4	
0	18	NA18558 NA18562 NA18573 NA18947 NA18943 NA18991
1	20	NA18532 NA18990 NA18572 NA18621 NA18636 NA18976 NA18968 NA18573
2	23	NA18980 NA18564 NA18542 NA18572 NA18974 NA18555 NA19000 NA18558
3	28	NA18552 NA18942 NA18562 NA18542 NA18563 NA18576 NA18965 NA18637 NA18967 NA18948 [illegible]
4	36	NA18997 NA18537 NA18964 NA18550 NA18971 NA18561 NA18940 NA18571 NA18987 NA18582 [illegible]
5	67	NA18605 NA18570 NA18611 NA18592 NA18998 NA18956
6	73	
18	5	
0	18	NA18579 NA18973
1	17	NA18564 NA18558 NA18635 NA18579
2	18	NA18571 NA18564 NA18603 NA18577 NA18624 NA18940 NA18951 NA18969
3	19	NA18545 NA18948 NA18552 NA18990 NA18577 NA18603
4	22	NA18562 NA18577 NA18572 NA18961 NA18636 NA18552 NA18967 NA18999 NA18973 NA18994
5	25	NA18561 NA18973 NA18564 NA18562
6	26	NA18532 NA18571 NA18592 NA18564 NA18943 NA18524 NA18994 NA18561
7	30	NA18579 NA18592 NA18973 NA18636
8	29	NA18620 NA18526 NA18965 NA18975 NA18552 NA18632 NA18609 NA18579 NA18636 NA18973 [illegible]
9	38	NA18612 NA18529 NA18971 NA18542 NA18980 NA18573 NA18953 NA18593 NA18555 NA18594 [illegible]
10	71	NA18949 NA18576 NA18966 NA18947
11	75	
19	1	
0	14	NA18577 NA18632 NA18593 NA18633
1	16	NA18956 NA18967
2	16	NA18967 NA18635
3	17	NA18579 NA18524 NA18635 NA18956 NA18966 NA18593

4	18	NA18624 NA18966 NA18636 NA18960 NA18994 NA18623
5	23	NA18943 NA18940 NA18524 NA18980 NA18532 NA18526 NA18608 NA18577 NA18945 NA18579 NA1
6	30	NA18969 NA18537 NA18620 NA18550 NA18970 NA18555 NA18975 NA18561 NA18995 NA18573 NA1
7	63	NA18947 NA18529 NA19007 NA18542 NA18603 NA18563 NA18956 NA18571 NA18968 NA18609 NA1
8	83	
19	2	
0	14	NA18621 NA18968 NA18995 NA18537
1	16	NA18633 NA18991 NA18968 NA18621
2	16	NA18526 NA18968
3	16	NA18636 NA18555 NA18997 NA18526
4	16	NA18545 NA18636 NA18971 NA18997
5	16	NA18577 NA18545
6	17	NA18573 NA18969 NA18636 NA18577
7	18	NA18577 NA18636 NA18959 NA18633 NA18965 NA18971
8	19	NA18624 NA18592 NA18570 NA18959 NA18636 NA18577 NA18942 NA18637
9	22	NA18545 NA18978 NA18611 NA18624 NA18940 NA18579 NA18964 NA18609 NA18973 NA18570
10	26	NA18635 NA18524 NA18967 NA18972 NA18582 NA18961 NA18624 NA18611 NA18971 NA18944 NA
11	38	NA18564 NA18532 NA18620 NA18542 NA18612 NA18550 NA18949 NA18552 NA18563 NA18558 NA
12	66	NA18999 NA18623
13	68	
19	3	
0	14	NA18577 NA18605 NA18594 NA18566 NA18609 NA18960 NA18942 NA18997
1	16	NA18635 NA18594 NA18637 NA18953 NA18959 NA18542
2	19	NA18532 NA18572 NA18620 NA18612 NA18624 NA18609 NA18969 NA18981
3	25	NA18995 NA18537 NA18552 NA18624 NA18555 NA18637 NA18636 NA18999 NA18943 NA18976 NA1
4	38	NA18991 NA18526 NA18971 NA18545 NA18968 NA18550 NA18970 NA18563 NA18579 NA18576 NA1
5	73	NA18965 NA18623
6	75	
19	4	
0	14	NA18537 NA18532 NA18558 NA18566 NA18978 NA18972
1	15	NA18570 NA18577 NA18573 NA18947 NA18633 NA18537 NA18964 NA18558
2	16	NA18563 NA18570
3	16	NA18558 NA18964 NA18577 NA18563
4	17	
19	5	
0	14	NA18526 NA18561 NA18624 NA18632 NA18949 NA18612 NA18987 NA18999
1	17	NA18524 NA18603 NA18552 NA18526 NA18612 NA18949 NA18968 NA18998
2	18	NA18563 NA18552 NA18636 NA18524
3	19	NA18524 NA18636 NA18532 NA18968 NA18552 NA18563
4	18	NA18563 NA18552 NA18582 NA18592 NA18636 NA18524
5	18	NA18524 NA18636 NA18561 NA18563 NA18577 NA18532
6	18	NA18552 NA18561 NA18975 NA18612
7	18	NA18561 NA18552 NA18636 NA18524 NA18969 NA18975
8	19	NA18524 NA18636 NA18566 NA18561 NA18609 NA18951 NA18991 NA18582
9	18	NA18526 NA18566 NA18558 NA18593 NA18636 NA18524 NA18637 NA18609 NA18978 NA18969
10	21	NA18942 NA18563 NA18947 NA18636 NA18960 NA18526
11	21	NA18561 NA18960 NA18612 NA18947 NA18967 NA19005
12	23	NA18524 NA18612 NA18972 NA18956
13	25	NA18623 NA18524 NA18951 NA18978 NA18974 NA18561 NA18994 NA18637
14	28	NA18570 NA18948 NA18526 NA18974 NA18635 NA18991 NA18636 NA18623 NA18637 NA18951 NA
15	34	NA18572 NA18564 NA18573 NA18571 NA18953 NA18594 NA18970 NA18945 NA18943 NA18952 NA
16	55	NA18944 NA18537 NA19007 NA18576 NA18971 NA18611 NA18966 NA18940
17	63	
20	1	

```
0         10
20         2
0         10         NA18532 NA18623 NA18609 NA18975 NA18973 NA18961
1         12         NA18571 NA18973 NA18632 NA18609
2         12         NA18579 NA18632 NA18971 NA18571
3         12         NA18632 NA18579 NA18637 NA18999
4         12         NA18561 NA18632
5         12         NA18564 NA18637 NA18972 NA18561
6         12         NA18561 NA18972
7         12         NA18579 NA18561 NA18609 NA18558 NA18953 NA18564
8         12         NA18561 NA18552 NA18571 NA18971 NA18611 NA18579 NA18966 NA19005
9         12
20         3
0         10         NA18582 NA18592 NA18975 NA18964 NA19000 NA18633
1         10         NA18592 NA19000
2         10         NA18603 NA18621
3         10         NA18964 NA18603
4         10         NA18949 NA18592 NA18970 NA18964
5         10         NA18592 NA18949 NA18964 NA18970 NA18978 NA18965
6         10         NA18555 NA18964
7         10         NA18532 NA18555
8         10         NA18603 NA18532
9         10         NA18566 NA18579 NA18576 NA18603
10         10         NA18545 NA18576 NA18949 NA18592
11         10         NA18532 NA18545
12         10         NA18555 NA18975 NA18571 NA18532
13         10         NA18532 NA18571 NA18592 NA18949
14         10         NA18570 NA18532
15         10         NA18532 NA18570
16         10         NA18570 NA18532
17         10         NA18532 NA18570
18         10         NA18570 NA18532
19         10         NA18532 NA18570
20         10         NA18570 NA18532
21         10         NA18532 NA18570
22         10         NA18570 NA18532
23         10         NA18532 NA18570
24         10         NA18570 NA18532
25         10         NA18532 NA18570
26         10         NA18570 NA18532
27         10         NA18532 NA18570
28         10         NA18570 NA18532
29         10         NA18532 NA18570
30         10         NA18570 NA18532
20         4
0         10         NA18542 NA18945
1         10         NA18545 NA18542 NA18965 NA18609
2         10         NA18961 NA18545 NA18991 NA18971
3         8         NA18603 NA18636 NA18987 NA18991
4         8         NA18582 NA18942
5         8         NA18945 NA18961 NA18969 NA18582
6         7         NA18537 NA18969 NA18608 NA18965 NA18961 NA18945
7         7         NA18550 NA18974 NA18965 NA18608 NA18969 NA18537
8         6         NA18612 NA18603 NA18991 NA18969
```

```
9         6         NA18636 NA18991
10         6
20          5
0         10         NA18537 NA18529
1         10         NA18577 NA18537 NA18624 NA18944
2         11         NA18529 NA18971 NA18976 NA18550
3         11         NA18558 NA18529 NA18608 NA18635 NA18942 NA18978
4         11         NA18991 NA18577
5         10         NA18577 NA18991
6         11         NA18991 NA18577
7         10         NA18577 NA18991
8         11         NA18991 NA18577
9         10         NA18577 NA18991
10         11         NA18991 NA18577
11         10         NA18577 NA18991
12         11         NA18991 NA18577
13         10         NA18577 NA18991
14         11         NA18991 NA18577
15         10         NA18577 NA18991
16         11         NA18991 NA18577
17         10         NA18577 NA18991
18         11         NA18991 NA18577
19         10         NA18577 NA18991
20         11         NA18991 NA18577
21         10         NA18577 NA18991
22         11         NA18991 NA18577
23         10         NA18577 NA18991
24         11         NA18991 NA18577
25         10         NA18577 NA18991
26         11         NA18991 NA18577
27         10         NA18577 NA18991
28         11         NA18991 NA18577
29         10         NA18577 NA18991
30         11         NA18991 NA18577
```

> *myresoutput*

```
      [,1] [,2] [,3] [,4] [,5]
 [1,]    2    2    2    2    2
 [2,]    2    2    2    2    2
 [3,]    2    2    2    2    2
 [4,]    2    2    2    2    2
 [5,]    2    2    2    2    2
 [6,]    2    2    2    2    2
 [7,]    2    2    2    2    2
 [8,]    2    2    2    2    2
 [9,]    2    2    2    2    2
[10,]    2    2    2    2    2
[11,]    2    2    3    2    3
[12,]    3    3    3    3    2
[13,]    3    3    3    3    3
[14,]    3    3    3    3    3
[15,]    3    3    3    3    3
[16,]    3    3    4    3    4
[17,]    6    4    4    4    5
```

```
[18,]    9    6    8    7   12
[19,]    9   14    7    5   18
[20,]    1   10   31   11   31
```

```
> myunrescount
```

```
      [,1] [,2] [,3] [,4] [,5]
 [1,]    0    0    0    0    0
 [2,]    0    0    0    0    0
 [3,]    0    0    0    0    0
 [4,]    0    0    0    0    0
 [5,]    0    0    0    0    0
 [6,]    0    0    0    0    0
 [7,]    0    0    0    0    0
 [8,]    0    0    0    0    0
 [9,]    0    0    0    0    0
[10,]    0    0    0    0    0
[11,]    0    0    0    0    0
[12,]    0    0    0    0    0
[13,]    0    0    0    0    0
[14,]    0    0    0    0    0
[15,]    0    0    3    0    4
[16,]    0    0    0    0    0
[17,]    9    9    4    3   10
[18,]   16   11   14   17   15
[19,]    7   22   15   73   27
[20,]   80   78   80   84   80
```

And now plot those results

```
> par(mar=c(5.1,4.1,1.1,4.1))
> plot(1-apply(myunrescount,1,sum,na.rm=T)/seq(20,400,20),axes=F,xlab="number of samples switched",ylab=
> points(apply(myresoutput,1,median,na.rm=T)/12,pch=16,col="red",type="b")
> axis(1,at=1:20,labels=seq(4,80,4),las=2)
> axis(2,at=seq(0,1,0.2),las=2)
> axis(4,at=seq(0,1,1/6),labels=seq(0,12,2),las=2)
> mtext("median number of iterations taken",side=4,line=2)
> box()
```

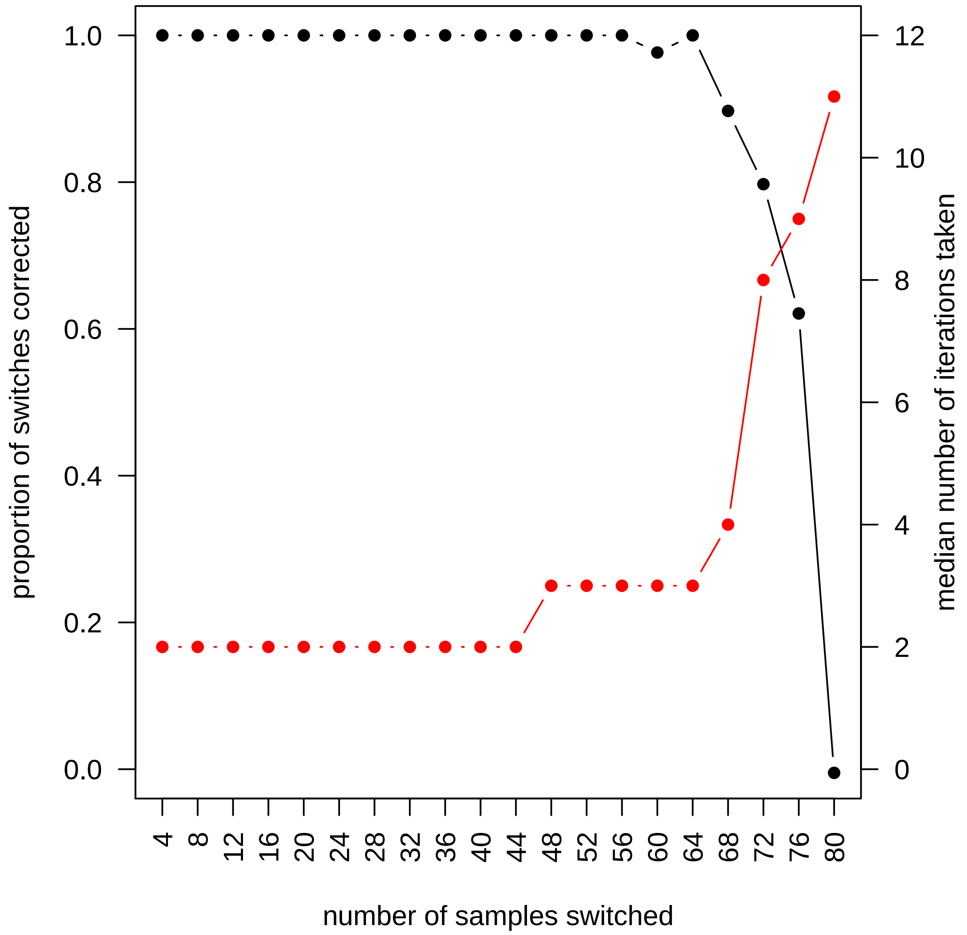

proportion of switches corrected
median number of iterations taken
number of samples switched
1.0
0.8
0.6
0.4
0.2
0.0
12
10
8
6
4
2
0
4
8
12
16
20
24
28
32
36
40
44
48
52
56
60
64
68
72
76
80
