# Supplementary material for: Calling Sample Mix-Ups in Cancer Population Studies
Source: PLoS One. 2012 Aug 9;7(8):e41815. doi: 10.1371/journal.pone.0041815 (PMC3415393; doi:10.1371/journal.pone.0041815)
Supplement: Sweave S2 — Sweave file [27] and supporting data to enable reproduction of analyses not relating to the ‘Close relatives and validation’ section of this article. (ZIP) [file pone.0041815.s002.zip › BADGER.pdf]

Code to reproduce results and figures in the paper "Calling sample mixups in cancer population studies"

Andy Lynch
Department of Oncology, University of Cambridge

1 Preparations

Due to the nature of the data analysed here, the values must be obtained from the METABRIC study. We give here template objects indicating the arrays and probes that need to be included in the matrices `MATEXP` and `MATSNP` in order that they can be filled out and the following code will run.

```
>   load("BADGERSWeaveData.Rda")
```

Note that when filled out, the top-left corner of MATEXP should look like...

```
> MATEXP[1:3,1:3]
             4660479011_H 4668171120_D          <NA>
ILMN_2228453     6.742111     7.050769            NA
ILMN_2273572     6.265458     7.585327            NA
ILMN_1726030     7.905480     8.549442            NA
```

... and the top-left corner of MATSNP should look like...

```
> MATSNP[1:3,1:3]
              A430_0077 A430_0007 A430_0019
SNP_A-2044454         2         1         1
SNP_A-4273613         1         2         2
SNP_A-2099552         2         3         3
```

Note also that 343 columns of the MATEXP matrix are empty, as are 14 columns of the MATSNP matrix, and that the modes of both are "numeric".

It is necessary to infer the table of quartets in the data. Those are the tumours for which both types of array were run for both the tumour and matched normal tissue.

```
> temp<-paper1mappings$ID[which( (paper1mappings$Type=="N") &
+   (!is.na(paper1mappings$IlluminaID)) )]
> temp<-temp[temp %in% paper1mappings$Known_matched_ID]
> quartets<-cbind(paper1mappings$ArraymatchID[match(temp,paper1mappings$Known_matched_ID)],
+                 paper1mappings$IlluminaID[match(temp,paper1mappings$Known_matched_ID)],
+                 paper1mappings$ArraymatchID[match(temp,paper1mappings$ID)],
```

```
+                  paper1mappings$IlluminaID[match(temp,paper1mappings$ID)])
> quartets<-quartets[!is.na(quartets[,3]),]
> dim(quartets)
```

```
[1] 127   4
```

```
> head(quartets)
```

```
     [,1]        [,2]            [,3]        [,4]
[1,] "A488_2560" "4668171060_C" "A488_1575" "5059343025_I"
[2,] "A430_0085" "4616494072_E" "A488_1576" "5056679043_A"
[3,] "A488_0061" "4668171081_C" "A488_1553" "5059343039_E"
[4,] "A488_0357" "4668171084_H" "A488_1577" "5059343040_F"
[5,] "A488_0003" "4668171063_K" "A488_1543" "5059343041_E"
[6,] "A488_0075" "4668171053_A" "A488_1520" "5059343038_J"
```

So we can see that there are 127 such quartets.

```
>   library(compiler)
```

We now define the BADGER functions. In general use, only the `BADGER()` function needs be called directly, but for this investigation we will be calling the component functions. `BADGER()` requires two matched matrices of expression and SNP values (`MATEXP` and `MATSNP`) from which to infer the eQTL relationships, a matrix of expression values for which to predict genotypes (`BADGEREXP`) and a matrix of observed SNPs (`BADGERSNP`) to which these predictions can be compared.
If either `BADGEREXP` or `BADGERSNP` are not provided, the appropriate matched matrix (`MATEXP` or `MATSNP`) is used instead. BADGER also takes a threshold (`RESCO`) used to filter the eQTLs based on the residuals of the predicted values amongst the matched arrays. The output is a matrix of scores comparing all expression arrays in `BADGEREXP` to all SNP arrays in `BADGERSNP`.

```
> BADGERpredSNP<-function(MATEXP,MATSNP,BADGEREXP){
+ if(is.null(BADGEREXP)){BADGEREXP<-MATEXP}
+   preds<-matrix(NA,nrow=nrow(BADGEREXP),ncol=ncol(BADGEREXP))
+         for(i in 1:(nrow(MATEXP))){
+                 #cat(i," ")
+                 dens1<-density((MATEXP)[i,which(MATSNP[i,]==1)],na.rm=T)
+                 dens2<-density((MATEXP)[i,which(MATSNP[i,]==2)],na.rm=T)
+                 dens3<-density((MATEXP)[i,which(MATSNP[i,]==3)],na.rm=T)
+                 for(j in 1:(ncol(BADGEREXP))){
+                         useval<-BADGEREXP[i,j]
+                         if(is.na(useval)){useval<-mean(MATEXP[i,],na.rm=T)}
+                         pred1<-dens1$y[which.min(abs(dens1$x-useval))]
+                         pred2<-dens2$y[which.min(abs(dens2$x-useval))]
+                         pred3<-dens3$y[which.min(abs(dens3$x-useval))]
+                         predsum<-pred1+pred2+pred3
+                         pred1<-pred1/predsum
+                         pred2<-pred2/predsum
+                         pred3<-pred3/predsum
+                         preds[i,j]<-(pred1+2*pred2+3*pred3)
```

```
+                 }
+         }
+         colnames(preds)<-colnames(BADGEREXP)
+         return(preds)
+ }
> B.predSNP<-cmpfun(BADGERpredSNP)
> BADGERmatchScores<-function(predictedSNP,BADGERSNP,useEQTL=NULL){
+         if(is.null(useEQTL)){useEQTL<-1:(nrow(BADGERSNP))}
+         matchscore<-matrix(NA,nrow=ncol(predictedSNP),ncol=ncol(BADGERSNP))
+                 for(pred in 1:ncol(predictedSNP)){
+                         for(obs in 1:ncol(BADGERSNP)){
+                                 matchscore[pred,obs]<-sum((predictedSNP[useEQTL,pred]-
+                                 BADGERSNP[useEQTL,obs])^2,na.rm=T)
+                         }
+                 }
+         return(matchscore)
+         }
> B.MS<-cmpfun(BADGERmatchScores)
> QTLres<-function(predictedMATSNP,MATSNP){
+         matchscore<-rep(NA,nrow(MATSNP))
+                 for(EQTL in 1:nrow(MATSNP)){
+                         matchscore[EQTL]<-sum((predictedMATSNP[EQTL,]
+                              -MATSNP[EQTL,])^2,na.rm=T)
+                 }
+         return(matchscore)
+         }
> B.RES<-cmpfun(QTLres)
> BADGER<-function(MATEXP,MATSNP,BADGEREXP=NULL,BADGERSNP=NULL,RESCO=NULL){
+ filtpreds<-B.predSNP(MATEXP,MATSNP)
+ RES<-B.RES(filtpreds,MATSNP)
+ if(is.null(RESCO)){RESCO<-(max(RES)+1)}
+ if(is.null(BADGEREXP)){preds<-filtpreds}
+   else{preds<-B.predSNP(MATEXP,MATSNP,BADGEREXP)}
+ if(is.null(BADGERSNP)){
+   MS<-B.MS(preds,MATSNP,useEQTL=which(RES<RESCO))
+   colnames(MS)<-colnames(MATSNP)
+   }
+   else{
+   MS<-B.MS(preds,BADGERSNP,useEQTL=which(RES<RESCO))
+   colnames(MS)<-colnames(BADGERSNP)
+   }
+ rownames(MS)<-colnames(preds)
+ MS
+ }
```

2 BADGER demonstration

While we have matched SNP and expression arrays already we only wish to use the tumours to predict the eQTL behaviour.

```
> predictedSNP<-B.predSNP(MATEXP[,which(paper1mappings$Type=="T")],
+                          MATSNP[,which(paper1mappings$Type=="T")],
+                          MATEXP)

> eQTLres<-B.RES(predictedSNP,MATSNP)
> table(eQTLres<1100)

FALSE  TRUE
  258   125

> hist(eQTLres,breaks=seq(200,2000,100))
> abline(v=1100,col="red")
```

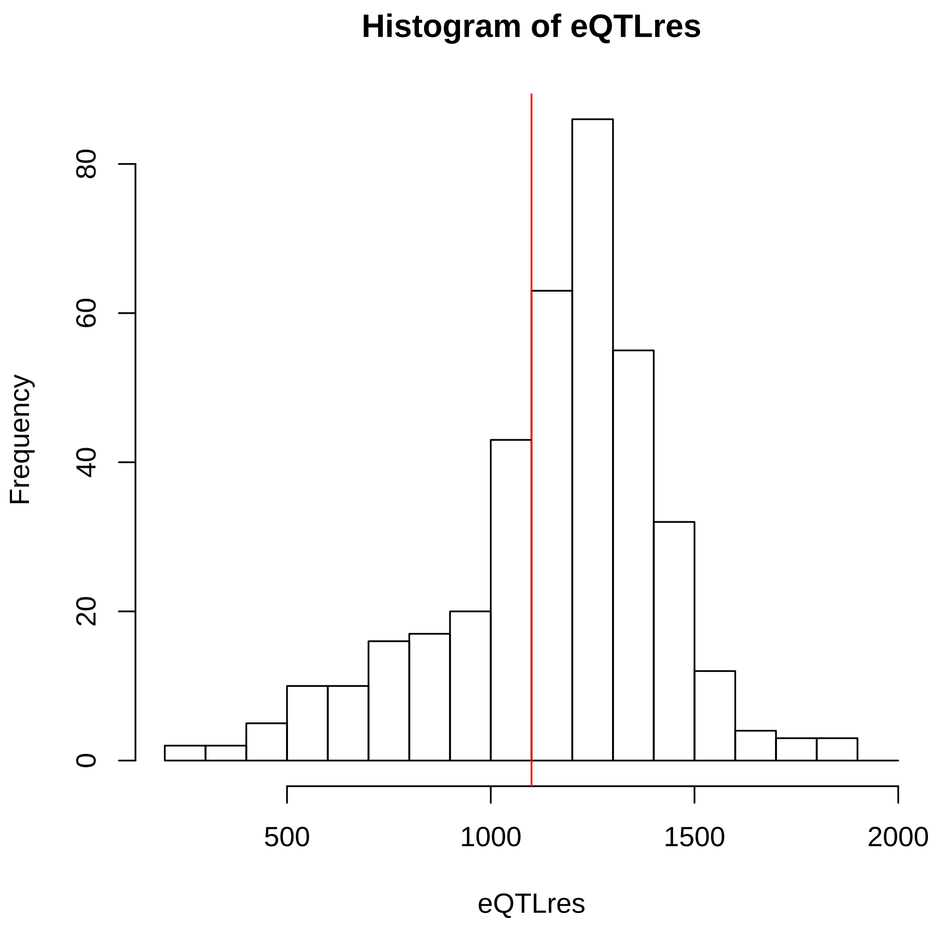

Now generate the scores for every expression-array:SNP-array, but note that we need to blank out the rows associated with non-existant arrays. We also generate matrices of the column ranks and row ranks of these scores

```
> matchscore<-B.MS(predictedSNP,MATSNP,useEQTL=which(eQTLres<1100))
> matchscore[is.na(paper1mappings$IlluminaID),]<-NA
> matchscore[,is.na(paper1mappings$ArraymatchID)]<-NA
> matchrank<-matrix(NA,nrow=nrow(matchscore),ncol=ncol(matchscore))
> for(i in 1:nrow(matchscore)){
+ matchrank[i,]<-rank(matchscore[i,],ties.method="min")
+ }
> matchrank[is.na(paper1mappings$IlluminaID),]<-NA
> matchrank[,is.na(paper1mappings$ArraymatchID)]<-NA
> matchSNPrank<-matrix(NA,nrow=nrow(matchscore),ncol=ncol(matchscore))
> for(i in 1:ncol(matchscore)){
+ matchSNPrank[,i]<-rank(matchscore[,i],ties.method="min")
+ }
> matchSNPrank[is.na(paper1mappings$IlluminaID),]<-NA
> matchSNPrank[,is.na(paper1mappings$ArraymatchID)]<-NA
```

Since our original matrices were matched, our matchscore matrix is square, and the ranks along the diagonal should be low. If we tabulate the diagonal of that matrix, we see that the vast majority are indeed low, with just a dozen or so matches that require investigation.

```
> table(diag(matchrank))

   1    2    3    4    5    6    7   10   11   13   14   15   23   25   35   77
1846  234   14    8    2    3    1    2    1    1    1    1    2    1    1    1
 120  256  665
   1    1    1

> which(pmin(diag(matchrank),diag(matchSNPrank))>5)

[1]    6   41   42   44   71   77  159 1345 1388
```

3 Numbers for Figure 3

We need to extract the elements of the matchrank matrix corresponding to the quartets.

```
> ComparisonR<-matrix(ncol=4,nrow=dim(quartets)[1])
> for(i in 1:dim(quartets)[1]){
+ TS<-match(quartets[i,1],paper1mappings$ArraymatchID)
+ NS<-match(quartets[i,3],paper1mappings$ArraymatchID)
+ TE<-match(quartets[i,2],paper1mappings$IlluminaID)
+ NE<-match(quartets[i,4],paper1mappings$IlluminaID)
+ ComparisonR[i,1]<-matchrank[TE,TS]
+ ComparisonR[i,2]<-matchrank[TE,NS]
+ ComparisonR[i,3]<-matchrank[NE,TS]
+ ComparisonR[i,4]<-matchrank[NE,NS]
+ }
> cat("top left \n")

top left
```

```
> table(ComparisonR[,2])

  1   2
104  23

> sum(ComparisonR[,2]<ComparisonR[,1])

[1] 64

> cat("bottom left \n")

bottom left

> table(ComparisonR[,1])

 1  2  4
63 63  1

> sum(ComparisonR[,1]<ComparisonR[,2])

[1] 23

> cat("top right \n")

top right

> table(ComparisonR[,4])

  1   2   4
111  15   1

> sum(ComparisonR[,4]<ComparisonR[,3])

[1] 71

> cat("bottom right \n")

bottom right

> table(ComparisonR[,3])

 1  2  3 44
55 69  2  1

> sum(ComparisonR[,3]<ComparisonR[,4])

[1] 16
```

4 Generate Figure 5

```
> #pdf("Fig5.pdf",height=7.5,width=15)
> par(mfrow=c(1,2))
> boxplot(apply(matchscore[paper1mappings$Type=="T",],1,mean,na.rm=T)
+         ~paper1mappings$Eth[paper1mappings$Type=="T"],
+         ylim=c(70,110),col="grey70",ylab="Mean BADGER score",
+         names=c("Africa","Africa/Europe","Asia","Europe","Europe/Asia"))
> axis(3,at=1:5,labels=paste(rep("n=",5),table(paper1mappings$Eth),sep=""))
> boxplot(apply(matchscore[paper1mappings$Type=="T",],1,min,na.rm=T)
+         ~paper1mappings$Eth[paper1mappings$Type=="T"],
+         ylim=c(20,70),col="grey70",ylab="Minimum BADGER score",
+         names=c("Africa","Africa/Europe","Asia","Europe","Europe/Asia"))
> axis(3,at=1:5,labels=paste(rep("n=",5),table(paper1mappings$Eth),sep=""))
> #dev.off()
```

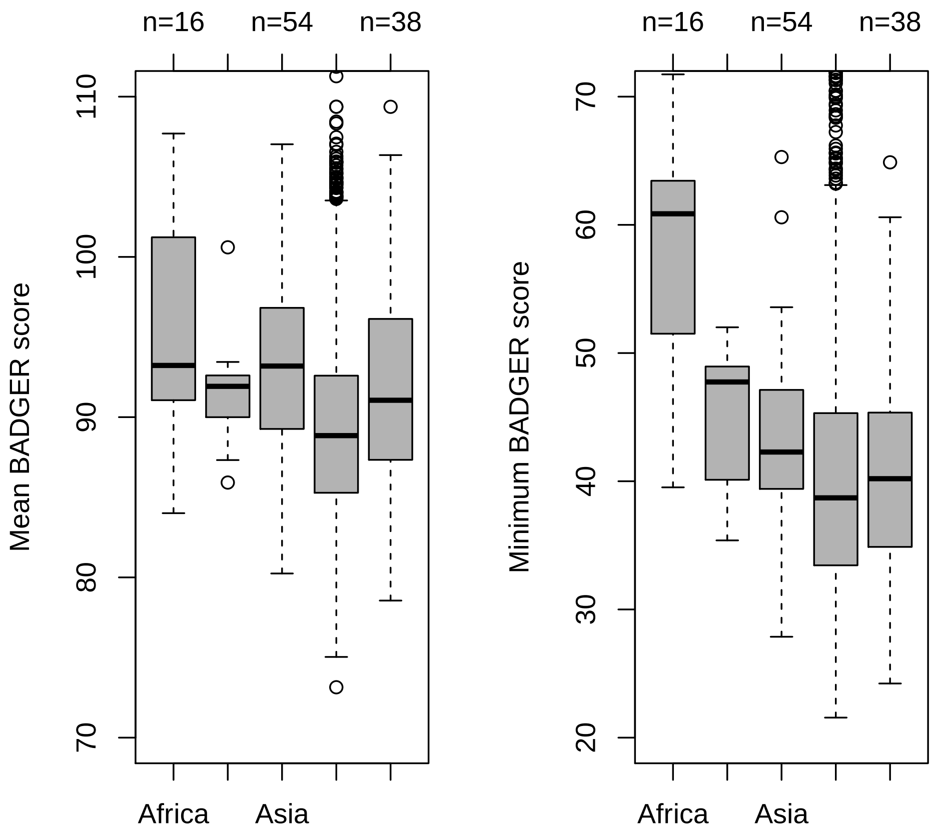

5 Generate Figure 6

```
> #pdf("Fig6.pdf",height=7.5,width=15)
> k<-202
> par(mfrow=c(1,3))
> boxplot(MATEXP[k,paper1mappings$Eth=="Europe Group"]
+         ~MATSNP[k,paper1mappings$Eth=="Europe Group"],
+         ylim=c(6,12),xlab="Genotype",
+         ylab="Expression",main="Europe",col="grey70")
> temp<-table(MATSNP[k,paper1mappings$Eth=="Europe Group"])
> axis(3,at=1:length(temp),labels=paste(rep("n=",length(temp)),temp,sep=""),tick=F,line=-0.5)
> boxplot(MATEXP[k,paper1mappings$Eth=="Asia Group"]
+         ~MATSNP[k,paper1mappings$Eth=="Asia Group"],
+         ylim=c(6,12),xlab="Genotype",
+         ylab="Expression",main="Asia",col="grey70")
> temp<-table(MATSNP[k,paper1mappings$Eth=="Asia Group"])
> axis(3,at=1:length(temp),labels=paste(rep("n=",length(temp)),temp,sep=""),tick=F,line=-0.5)
> boxplot(MATEXP[k,paper1mappings$Eth=="Africa Group"]
+         ~MATSNP[k,paper1mappings$Eth=="Africa Group"],
+         ylim=c(6,12),xlab="Genotype",
+         ylab="Expression",main="Africa",col="grey70")
> temp<-table(MATSNP[k,paper1mappings$Eth=="Africa Group"])
> axis(3,at=1:length(temp),labels=paste(rep("n=",length(temp)),temp,sep=""),tick=F,line=-0.5)
> #dev.off()
```

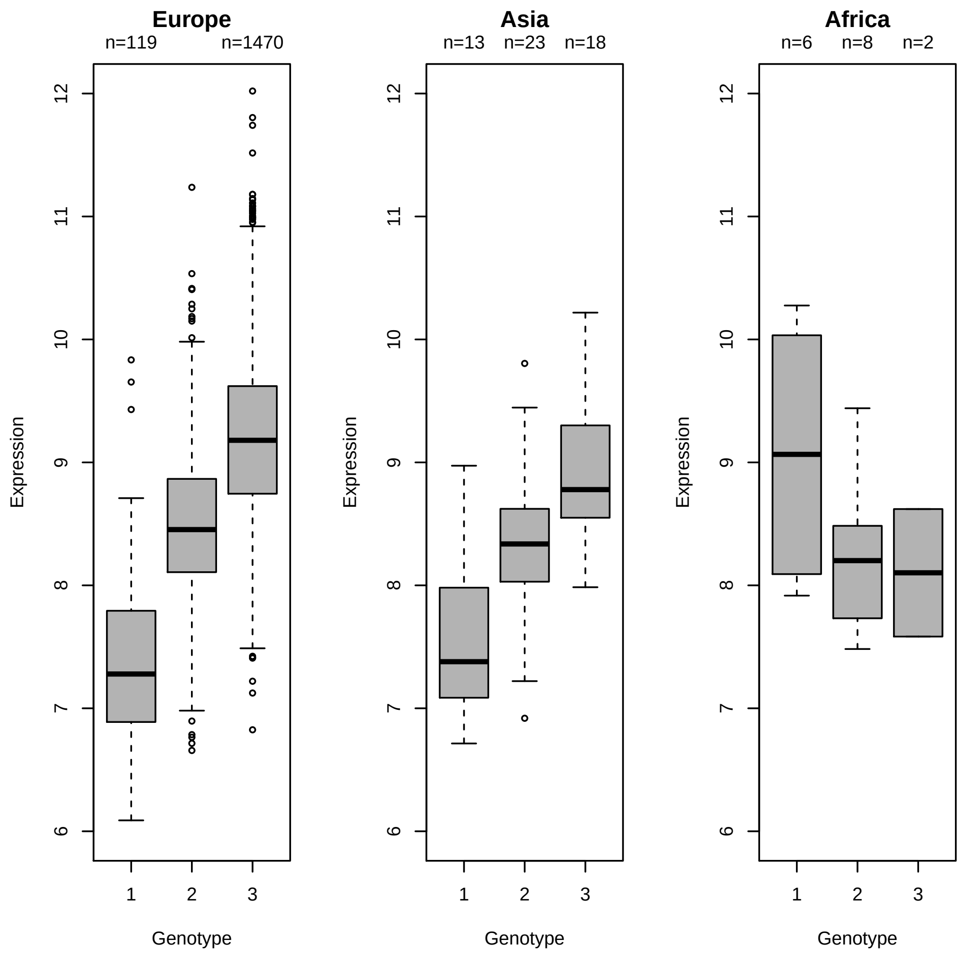

6 SNP bias in selected SNPs

```
> updateSNP<-updateSNP[match(rownames(MATEXP),updateSNP[,1]),]
> SNPnum<-updateSNP$SNP_number
> SNPnum[SNPnum>3]<-4
> table(SNPnum,eQTLres<1100)

SNPnum FALSE TRUE
     0   147   52
     1    80   39
     2    20   18
     3     5    8
     4     6    8
```

7 Figure 7

```
> EURQTLres<-B.RES(predictedSNP[,paper1mappings$Eth=="Europe Group"],
+                  MATSNP[,paper1mappings$Eth=="Europe Group"])
```

```
> AFRQTLres<-B.RES(predictedSNP[,paper1mappings$Eth=="Africa Group"],
+                   MATSNP[,paper1mappings$Eth=="Africa Group"])
> AISQTLres<-B.RES(predictedSNP[,paper1mappings$Eth=="Asia Group"],
+                   MATSNP[,paper1mappings$Eth=="Asia Group"])
> #pdf("Fig7.pdf",height=7.5,width=15)
> par(mfrow=c(1,2))
> boxplot((EURQTLres/2301)~SNPnum,names=c("0","1","2","3",">3"),
+         xlab="number of SNPS",ylab="mean squared residual",
+         main="Europe Group",col="grey70",ylim=c(0.2,0.7))
> temp<-table(SNPnum)
> axis(3,at=1:length(temp),labels=paste(rep("n=",length(temp)),temp,sep=""),tick=F,line=-0.5)
> boxplot((AFRQTLres/16)~SNPnum,names=c("0","1","2","3",">3"),
+         xlab="number of SNPS",ylab="mean squared residual",
+         main="Africa Group",col="grey70",ylim=c(0.2,0.7))
> axis(3,at=1:length(temp),labels=paste(rep("n=",length(temp)),temp,sep=""),tick=F,line=-0.5)
> #  dev.off()
```

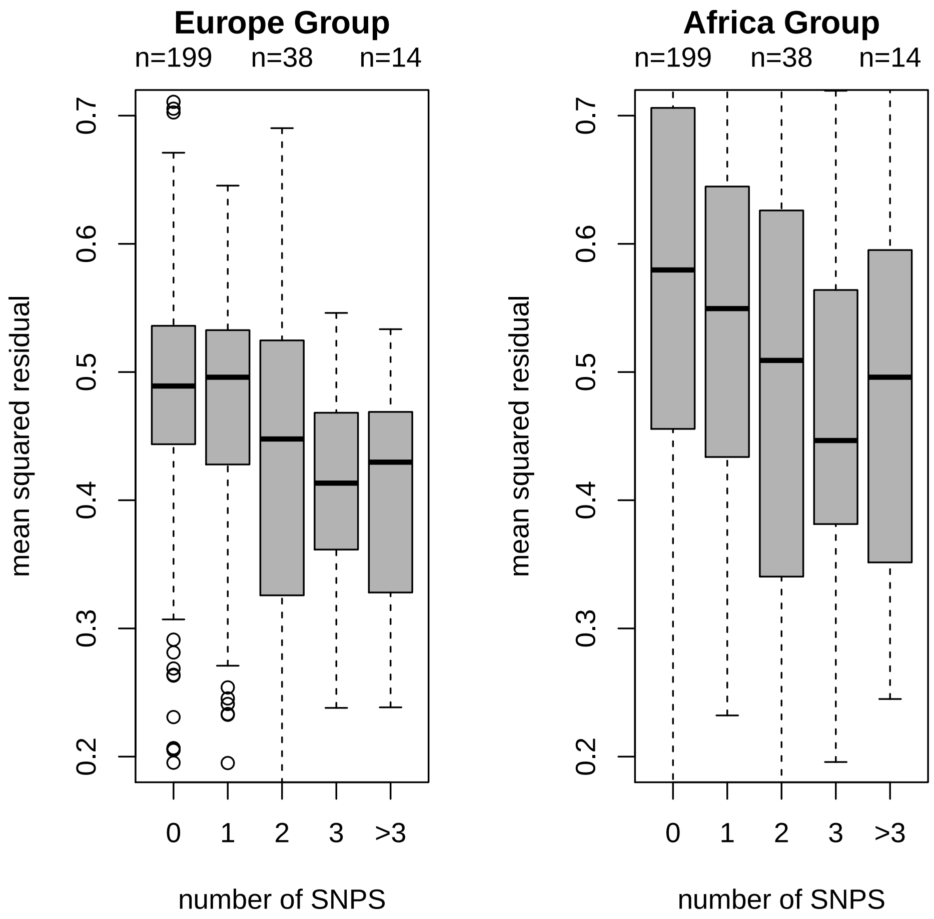

8 Design of Experiment numbers

```
> numberMissed<-function(x){
+ sumrandN<-rep(0,x)
+ for(j in 1:x){
+ temp<-matrix(sample(c(rep(1,48),rep(2,48)),96),nrow=8)
+ sumrandN[j]<-sum(temp[1:8,1:11]==temp[1:8,2:12])+sum(temp[1:7,1:12]==temp[2:8,1:12])
+ }
+ sumrandN
+ }
> c.nM<-cmpfun(numberMissed)
> sumrandN<-c.nM(100000)
> cat("number of simple switches we'll miss with a random layout")

number of simple switches we'll miss with a random layout

> mean(sumrandN)

[1] 85.08843

> quantile(sumrandN,c(0.025,0.975))

 2.5% 97.5%
   72    98

> platediffs<-function(x){
+ mindiffs<-rep(0,x)
+ for(k in 1:x){
+ listtemp<-array(data=NA,dim=c(8,12,100))
+ for(j in 1:100){
+ listtemp[,,j]<-matrix(sample(c(rep(1,48),rep(2,48)),96),nrow=8)
+ }
+ diffs<-matrix(NA,ncol=100,nrow=100)
+ for(j in 1:99){
+ for(i in (j+1):100){
+ diffs[i,j]<-sum(abs(listtemp[,,j]-listtemp[,,i]))
+ }
+ }
+ mindiffs[k]<-min(diffs,na.rm=T)/2
+ }
+ mindiffs
+ }
> c.pd<-cmpfun(platediffs)
> mindiffs<-c.pd(1000)
> cat("minimum number of differences between two of 100 arrays with a random layout")

minimum number of differences between two of 100 arrays with a random layout

> mean(mindiffs)
```

```
[1] 15.119

> quantile(mindiffs,c(0.025,0.975))

 2.5% 97.5% 
   13    16 
```
